# Coexistence of Photosynthetic Marine Microorganisms, Viruses and Grazers: Towards Integration in Ocean Ecosystem Models

**DOI:** 10.1111/1462-2920.70295

**Published:** 2026-04-16

**Authors:** Paul Frémont, Stephen J. Beckett, David Demory, Eric Carr, Christopher L. Follett, Debbie Lindell, David Talmy, Stephanie Dutkiewicz, Joshua S. Weitz

**Affiliations:** ^1^ Department of Biology University of Maryland College Park Maryland USA; ^2^ Institute for Health Computing University of Maryland North Bethesda Maryland USA; ^3^ Sorbonne Université, CNRS, UMR8176, Laboratoire de Biodiversité et Biotechnologies Microbiennes (LBBM) Observatoire Océanologique Banyuls‐sur‐Mer France; ^4^ Department of Microbiology University of Tennessee Knoxville Tennessee USA; ^5^ Department of Earth, Ocean and Ecological Sciences University of Liverpool Liverpool UK; ^6^ Faculty of Biology Technion—Israel Institute of Technology Haifa Israel; ^7^ Department of Earth, Atmospheric, and Planetary Sciences Massachusetts Institute of Technology Cambridge Massachusetts USA; ^8^ Center for Sustainability Science and Strategy Massachusetts Institute of Technology Cambridge Massachusetts USA; ^9^ Department of Physics University of Maryland College Park Maryland USA

**Keywords:** biogeochemistry, coexistence, marine ecology, phytoplankton, virus, zooplankton

## Abstract

Photosynthetic microorganisms are responsible for primary production at the base of the marine food web and influence global biogeochemistry. Their growth is balanced by mortality processes, including zooplankton grazing and viral lysis. These predators coexist despite competing for the same microorganisms. Here, we develop a community model of photosynthetic microorganisms, grazers and viruses that incorporates elemental quotas and is suitable for ocean ecosystem models. We evaluate the extent to which coexistence is facilitated by: (i) explicit infected phytoplankton; (ii) heterogeneity in susceptibility to viral infection; and (iii) higher‐order mortality for the predators. We show a trade‐off between the virus latent period and virulence in facilitating coexistence. The latent period generates oscillations that reduce the growth rate of the free virus, promoting coexistence. Heterogeneity in susceptibility supports coexistence through resource partitioning, while higher‐order mortality widens the coexistence regime. The model outcomes are sensitive to viral life history traits, including the percentage of infected cells and the balance between virally‐ and zooplankton‐induced mortality. Leveraging algebraic model equilibria, we identify parameter combinations that yield realistic ecological properties in simplified epipelagic environments. Our models suggest that efforts to embed virus dynamics in ocean ecosystem models should include moderate to strong resistance to viral infection.

## Graphical abstract

Our study explores coexistence regimes of a virus and a zooplankton with a single phytoplankton for different model structures (left panel). The inclusion of an infected class and a resistant class is sufficient to generate coexistence regimes (middle panel). Using algebraic solutions, we optimise our model emphasising the importance of including viral resistance (right panel).

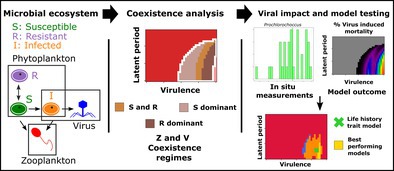

## 1 Introduction

Marine phytoplankton are a diverse group of photosynthetic microorganisms at the base of the ocean food web (Field et al. 1998) that play a crucial role in global biogeochemical cycles (Eppley and Peterson 1979) and are key contributors to the biological carbon pump (Henson et al. 2012; Guidi et al. 2015; Frémont et al. 2022). Phytoplankton are subject to multiple modes of mortality. One such mortality mode is predation/consumption by zooplankton such as heterotrophic nanoflagelattes (Zwirglmaier et al. 2009), dinoflagellates (Sherr and Sherr 1994) and copepods (Dussart 1965). Marine phytoplankton can also be infected by viruses (Suttle 2007), leading to the release of their cellular contents into the environment through cell lysis (Fuhrman and Noble 1995; Wilhelm and Suttle 1999; Weitz 2015). Viruses are present at high densities, typically exceeding $10^{10}/L$ in marine surface waters (Wigington et al. 2016) and can be major drivers of phytoplankton mortality (Bergh et al. 1989; Proctor and Fuhrman 1990; Fuhrman and Noble 1995; Suttle 2007; Mojica et al. 2016; Talmy, Beckett, Taniguchi, et al. 2019; Biggs et al. 2021; Carlson et al. 2022; Beckett et al. 2024) which, in turn, can lead to significant impacts on the flow of matter and nutrients (Wilhelm and Suttle 1999; Jover et al. 2014; Weitz et al. 2015; Guidi et al. 2015; Talmy, Beckett, Zhang, et al. 2019).

Current large‐scale ocean biogeochemistry models include various forms of biological complexity in their representation of biological and inorganic components (Ilyina et al. 2013; Aumont et al. 2015; Séférian et al. 2020; Stock et al. 2020; Negrete‐García et al. 2022). Some models incorporate comprehensive size‐structured food webs, featuring numerous classes of phytoplankton, diazotrophs, mixotrophs, and several types of grazers (Dutkiewicz et al. 2020; Stock et al. 2020). The increasing complexity of these models has allowed the theoretical exploration of numerous processes on a large scale, such as the emergence of global patterns of phytoplankton diversity (Dutkiewicz et al. 2020), the impact of ocean acidification (Dutkiewicz, Morris, et al. 2015), or the importance of iron in the limitation of phytoplankton growth (Tagliabue et al. 2016). However, these large‐scale models generally lack an explicit representation of the processes linked to phytoplankton infection by viruses, which has been recognised as an important future challenge (Mateus 2017). The parameterisation and implementation of such models remain challenging due to the complexity of virus‐host and virus‐environment interactions. Critically, this challenge requires both experiments characterising viral life history traits as well as in situ measurements of viral and phytoplankton concentrations to inform the models.

Datasets of experimental measurements of life history traits of phytoplankton viruses are increasingly available (Kirzner et al. 2016; Edwards and Steward 2018; Edwards et al. 2021; Maidanik et al. 2022). Measured traits include burst size (the number of virions produced in a single viral infection), latent period (the time between infection and viral lysis) and adsorption rate (the rate of encounter at which an infection is initiated). In particular, the adsorption rate of viruses can be highly variable and has been identified as crucial in determining viral population dynamics (Talmy, Beckett, Taniguchi, et al. 2019). This parameter depends both on the size of the host and the virus in relation to the size dependence of Brownian motion (Berg and Purcell 1977) and the swimming speed of host microbes (Murray and Jackson 1992; Talmy, Beckett, Taniguchi, et al. 2019; Edwards et al. 2021). For viruses, the adsorption rate can vary by several orders of magnitude and is typically less (sometimes significantly less) than biophysical limits (Talmy, Beckett, Taniguchi, et al. 2019). Efforts are also underway to harmonise the inference of viral life history traits by combining in vitro experiments with dynamical models of viral infection (Hinson et al. 2023; Dominguez‐Mirazo et al. 2024).

Likewise, in situ measurements of virus concentrations and infected cells (e.g., via the Polony (Baran et al. 2018; Goldin et al. 2020) and iPolony methods (Mruwat et al. 2021; Carlson et al. 2022)) are increasingly common and can be used to compare viral‐induced lysis to zooplankton grazing (Baudoux et al. 2007; Mojica et al. 2016; Carlson et al. 2022; Biggs et al. 2021; Beckett et al. 2024). For example, the observed infection percentage of *Prochlorococcus* cells is low in the surface waters of the North Pacific Subtropical gyre (NPSG) (Mruwat et al. 2021; Carlson et al. 2022), with cyanobacteria mortality dominated by zooplankton grazing (Beckett et al. 2024; Connell et al. 2020). However, significant latitudinal variation in the relative contribution to mortality by viruses and grazers has been shown (Mojica et al. 2016; Carlson et al. 2022). In the North Pacific, a hot spot of infection has been consistently reported over multiple years in the transition zone between subtropical and subpolar gyres (Carlson et al. 2022). In the southern ocean, high levels of cyanobacteria infection have also been reported in surface waters (Gochev et al. 2025). In the North Atlantic, virus‐induced mortality of cyanobacteria is an important factor at low and mid latitudes, while mortality associated with zooplankton dominated at higher latitudes ($>56^{{}^{\circ}}$ N) (Mojica et al. 2016). In eukaryotic phytoplankton, the grazing rates of *Phaeocystis* and picoeukaryotes have been shown to be dominated by viruses or zooplankton, but both rates were rarely found to be elevated simultaneously in the Southern Ocean (Biggs et al. 2021). These data suggest that there are cases where either viruses or zooplankton appear to be the dominant mortality agent, and their relative impact can vary depending on the eukaryotic phytoplankton taxon. For example, virally‐induced losses have been found to be more important in cryptophytes, while zooplankton grazing was found to be more important in diatoms (Biggs et al. 2021). Finally, in the northeastern oligotrophic Atlantic, viral‐induced mortality was the dominant process for picoeukaryote phytoplankton while zooplankton mortality was the dominant process for cyanobacteria (Baudoux et al. 2007).

Multiple studies have begun to integrate viral lysis into biogeochemical models, exploring their behaviour and investigating various questions related to the ecology and biogeochemical roles of viral infections in the oceans (Fuhrman 1999; Weitz et al. 2015; Talmy, Beckett, Zhang, et al. 2019; Talmy, Beckett, Taniguchi, et al. 2019; Biswas et al. 2020; Demory et al. 2020, 2021; Flynn et al. 2022; Beckett et al. 2024). From a biogeochemical perspective, viral lysis is hypothesised to increase organic matter recycling, increase net primary production and reduce energy transfer to higher trophic levels through reduced grazing by zooplankton, a set of processes known as the viral ‘shunt’ (Fuhrman 1999; Wilhelm and Suttle 1999; Weitz et al. 2015). In a modelling study of the California Current Ecosystem (Talmy, Beckett, Zhang, et al. 2019), it was estimated that a significant portion of the phytoplankton carbon loss from viral lysis is redirected into nutrient recycling through viral lysates. However, this estimate comes with considerable uncertainty (29% ± 20%) (Talmy, Beckett, Zhang, et al. 2019). It also remains unclear to what extent viral lysis could enhance carbon export through the viral ‘shuttle’, the process by which viral lysis products stimulate aggregation and sinking of particulate organic matter (POM), thereby enhancing carbon export to the deep ocean (Sullivan et al. 2017; Guidi et al. 2015; Zimmerman et al. 2020). In situ observations suggest that this is the case through aggregate formation resulting from cell lysis and faster sinking than individual cells (Yamada et al. 2018; Laber et al. 2018) and potentially through increased grazing on viral particles and infected cells (Zimmerman et al. 2020). Including viral lysis in large‐scale biogeochemical models would enable exploration of the relative balance of virus versus grazer impacts and ‘shunt’ and ‘shuttle’ mechanisms at global scales. Prior to incorporating viral lysis into large‐scale biogeochemical models, it is crucial to first develop and understand these ecological models in simpler settings. Doing so allows for a clearer understanding of virus, grazer and microbial dynamics.

A prerequisite to integration in large‐scale models, viral lysis models have to enable coexistence between the virus, the host and its zooplankton grazers. While a substantial body of literature explores the question of coexistence in ecological systems (Gause 1934; Hardin 1960; Armstrong and McGehee 1980; Chesson 1982; Polis and Holt 1992; Holt and Polis 1997; Huisman and Weissing 1999; Chesson 2000; Hin et al. 2011; Ellner et al. 2019; Yamamichi et al. 2022; Sieben et al. 2022; Orr et al. 2025), relatively few studies have examined how coexistence regimes emerge between viruses and zooplankton that both exploit phytoplankton as a resource (Levin et al. 1977; Weitz et al. 2015; Thingstad and Våge 2019; Biswas et al. 2020; Flynn et al. 2022). In the case of the Lotka–Volterra model (Lotka 1920), the competitive exclusion principle applies to a system with two predators and one prey where one predator excludes the other (Armstrong and McGehee 1980). In resource competition models increasing the complexity of the model by adding new resources can lead to chaotic behaviour, where coexistence can emerge from such dynamics, as demonstrated in the case of the paradox of the plankton (Huisman and Weissing 1999). Coexistence equilibria have also been investigated in host‐virus systems with the inclusion of an infected class and explicit nutrients (Levin et al. 1977), as well as in multitrophic marine food webs, including zooplankton grazing (Weitz et al. 2015). These studies suggest that fluctuating resource environments, endogenous oscillations and stabilising community effects can enable coexistence. Another theoretical study explored the role of selective grazing, where zooplankton avoid infected cells, in shaping the coexistence and dynamic behaviour (chaotic or stable) of a model that includes a grazer, a free virus, and an infected class of phytoplankton (Biswas et al. 2020). An adaptive host fitness model that responds to high viral abundance by increasing viral resistance has also been proposed to facilitate the coexistence between viruses, grazers and phytoplankton (Thingstad and Våge 2019). Finally, the inclusion of parasites, such as viruses, is expected to increase the coexistence within plankton communities and, consequently, the diversity of marine food webs (Thingstad 2000; Dunne et al. 2013; Weitz et al. 2015; Flynn et al. 2022).

**FIGURE 1:**
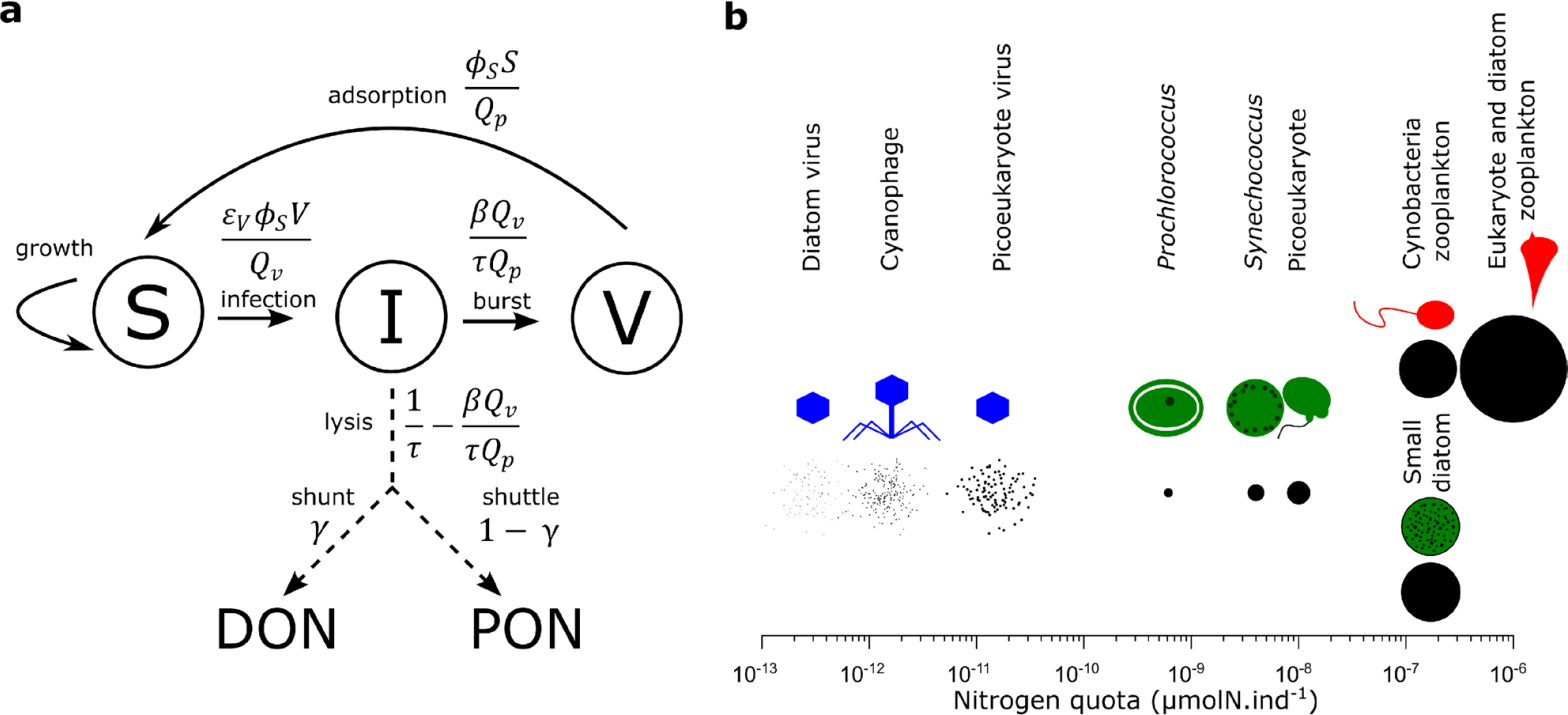
From the count SIV model to the elemental concentration SIV model. (a) Model sketch of the SIV elemental concentration model including a shunt and a shuttle term represented respectively as a flux (dashed arrows, not explicitly simulated) to the dissolved organic nitrogen (DON) and the particulate organic nitrogen pool (PON). DON and PON are not represented as explicit compartments here as we did not model their full dynamics. A fraction γ (respectively 1−γ) of the lysate is ‘shunted’ (‘shuttled’) to DON (to PON). The rates of matter fluxes (in $d^{-1}$) are presented for each arrow linked to the inclusion of the viral lysis component (the viral linear decay term is not represented here). (b) Nitrogen molar quotas of the different phytoplankton, their respective virus and grazer used in this study. Under the representation of each organism, the radius of each black circle is proportional to the cube root of the nitrogen molar quota, reflecting the scaling of nitrogen with cell volume. Each virus is shown 100 times for better visibility. The cyanobacteria morphology are inpired by *Prochlorococcus* and *Synechococcus*. The picoeukaryote morphology is inspired from *Micromonas pusilla* and the small diatom from *Minidiscus comicus*. Zooplankton are represented by a heterotrophic nanoflagellate for cyanobacteria grazing and by a heterotrophic dinoflagellate for eukaryotic phytoplankton grazing.

**FIGURE 2:**
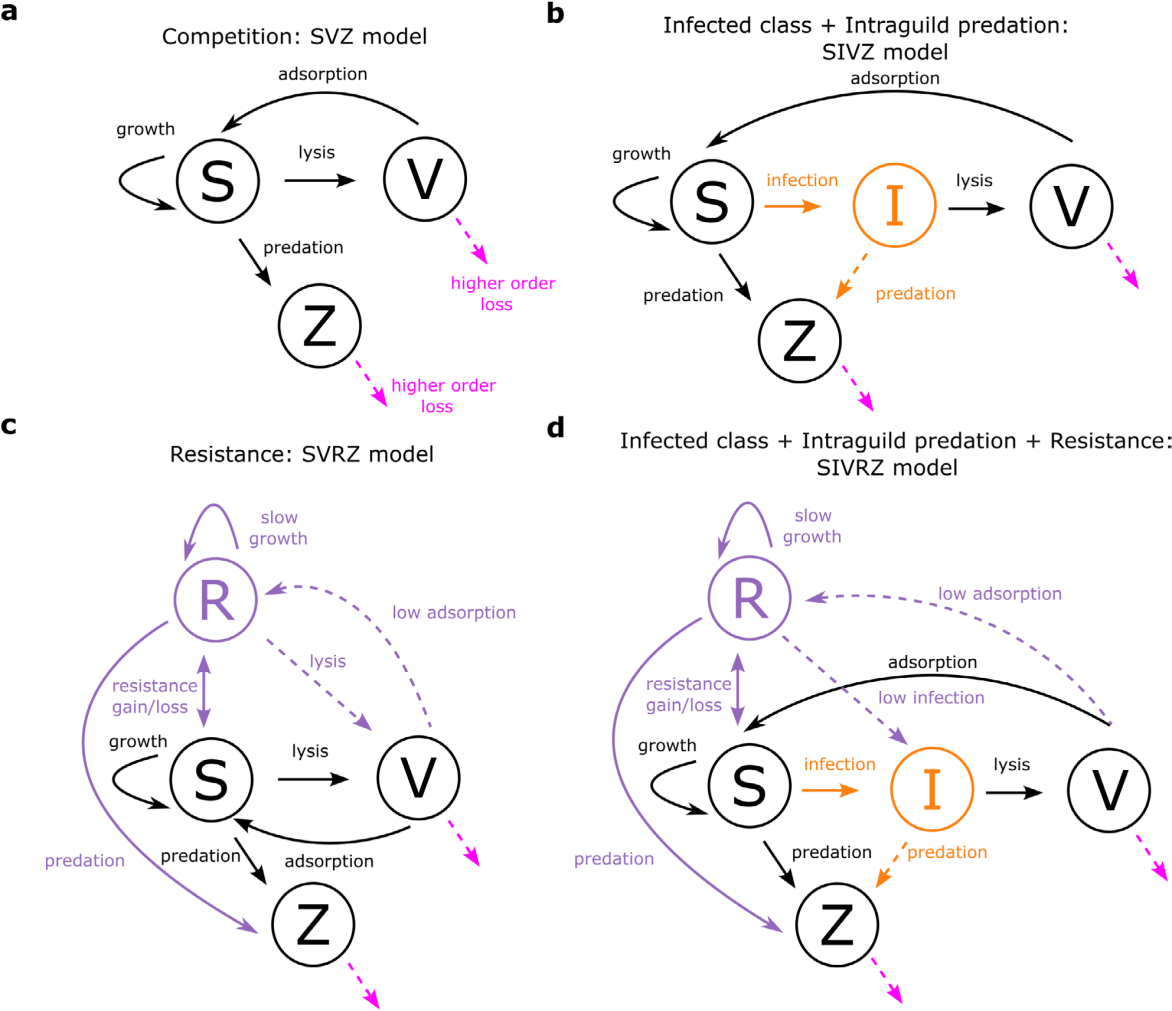
Model sketches illustrating model complexification to facilitate coexistence regimes between the virus and the zooplankton. Sketches of the different viral infection models used in the study: (a) SVZ model. (b) SVRZ model. (c) SIVZ model. (d) SIVRZ model. I, infected; R, resistant type; S, susceptible; V, virus; Z, zooplankton. Arrows represent fluxes of matter. Dashed arrows represent optional terms including higher order losses (quadratic mortality). Orange and purple colour respectively indicate fluxes linked to the addition of the I and R type.

In this study, we first develop an elemental quota version of the **S**usceptible cell—**I**nfected cell—**V**irus (SIV) model (Levin et al. 1977; Weitz 2015) which is suitable for large‐scale biogeochemical models accounting for cell and virion quotas. Including quotas provides a rationale for conversion between count and elemental concentration frameworks of viral ecology (Figure 1a). We develop extensions to this baseline model, including the addition of a grazing class, in order to explore how increasing ecological complexity (to include biologically relevant mechanisms) affects coexistence regimes for susceptible cells and their grazing and viral predators. We first explore the relevance of including an infected class (I) for the coexistence of the predators by comparing simplified models (SVZ and SIVZ, Figure 2a,b) across the virulence spectrum of the virus. Next, we incorporate mutations from the susceptible type (S) to an extracellular or intracellular resistant type in the SVRZ and SIVRZ models (Figure 2a,b), with varying resistance strengths, and examine the resulting coexistence regimes. Following this, we investigate the impact of including quadratic mortality terms, commonly used in biogeochemical models, for both viral and grazing predators. Coexistence analyses presented in the main text focus on *Prochlorococcus*, its phage, and a generic grazer, while analogous results for other phytoplankton types (*Synechococcus*, non‐diatom eukaryotes and diatoms) are provided in the Supporting Information. Finally, we define ecologically relevant concentrations and infection percentages, along with virus‐induced mortality rates, that the system is expected to reach for simplified oligotrophic and mesotrophic epipelagic environments and for four types of phytoplankton (Figure 1b). We then compare these values to model output and life history trait models, and discuss the empirical and theoretical relevance of our results to improving understanding of the joint role of viral and grazing predation to ocean ecosystems.

## 2 Materials and Methods

### 2.1 From the SIV Count Model to the SIV Elemental Concentration Model

We first convert the susceptible‐infected‐virus (SIV) count model (Levin et al. 1977; Weitz 2015), in units of individuals per volume (individual phytoplankton cell or virion), to an elemental concentration model, typically the currency of biogeochemical models (see Supporting Information for details of the calculation). Here, we arbitrarily chose units of nitrogen molar concentrations. In doing so, we write the viral ‘shunt’ as a flux of the resources from inside the phytoplankton cell to the dissolved organic nitrogen (DON) pool and the viral ‘shuttle’ as a flux to the particulate organic nitrogen (PON) pool but do not include the full microbial loop dynamics. The resulting SIV model for the virus‐phytoplankton dynamics (and lysate fluxes to dissolved and POM pools) can be written as follows:

(1a) $$\frac{\mathrm{dS}}{\mathrm{dt}}=\overset{\text{growth}}{\overbrace{\mu .S}}-\overset{\text{mortality}}{\overbrace{d_{S}.S}}-\overset{\text{successful infection}}{\overbrace{\frac{\epsilon_{V}.\phi_{S}}{Q_{v}}.S.V}}$$

(1b) $$\frac{\mathrm{dI}}{\mathrm{dt}}=\overset{\text{successful infection}}{\overbrace{\frac{\epsilon_{V}.\phi_{S}}{Q_{v}}.S.V}}-\overset{\text{mortality}}{\overbrace{d_{S}.I}}-\overset{\text{lysis}}{\overbrace{\frac{1}{\tau }.I}}$$

(1c) $$\frac{\mathrm{dV}}{\mathrm{dt}}=\overset{\text{vira lburst}}{\overbrace{\frac{\beta }{\tau }.\frac{Q_{v}}{Q_{p}}I}}-\overset{\text{viral decay}}{\overbrace{d_{V}.V}}-\overset{\text{adsorption}}{\overbrace{\frac{\phi_{S}}{Q_{p}}.S.V}}$$

(1d) $$J_{\mathrm{DON}}=\overset{\text{shunt}}{\overbrace{\gamma .\left(\frac{1}{\tau }-\frac{\beta }{\tau }.\frac{Q_{v}}{Q_{p}}\right).I}}$$

(1e) $$J_{\mathrm{PON}}=\overset{\text{shuttle}}{\overbrace{\left(1-\gamma \right).\left(\frac{1}{\tau }-\frac{\beta }{\tau }.\frac{Q_{v}}{Q_{p}}\right).I}}.$$

In the above, μ is the division rate of susceptible cells (S) in $d^{-1}$, $d_{S}$ is the cell mortality rate (natural cell death) in $d^{-1}$, $\phi_{S}$ is the adsorption rate of viruses to susceptible cells in $L.d^{-1}$, $\epsilon_{V}$ represents the inverse of intracellular resistance to the virus—that is the fraction of adsorption events that lead to a successful infection—and $d_{V}$ represents the decay rate of infectious virions in $d^{-1}$. Infections have an average duration of τ in d, denoting the latent period, after which a burst size, β, of new virions are released into the environment. $Q_{p}$ and $Q_{v}$ are the respective nitrogen quota of the phytoplankton and the virus in $\text{μmolN}.{\mathrm{ind}}^{-1}$. All tracers concentrations are in $\text{μmolN}.L^{-1}$. The fluxes to the dissolved and particulate pools, $J_{\mathrm{DON}}$ and $J_{\mathrm{PON}}$ (in $\text{μmolN}.L^{-1}.d^{-1}$) respectively, are included here for completeness. The microbial loop is not modelled explicitly but could be embedded as part of global ecosystem models (see Section 4 for further details). Assuming a fixed stoichiometry, Equations ((1a), (1b), (1c), (1d), (1e)) also applies to other elements (e.g., C or P) by substituting the nitrogen quota with the corresponding carbon or phosphorus quota, reflecting the different stoichiometry of viruses (Jover et al. 2014) and phytoplankton (Redfield 1934). We summarise the default set of parameters associated with all models in Tables S1–S3.

### 2.2 Elemental Concentration Models’ Equations: SVZ, SIVZ, SVRZ, SIVRZ

In this manuscript we explore four elemental concentration models of different complexity based on the above framework: the SVZ, SIVZ, SVRZ and SIVRZ models. These extensions also include a zooplankton, Z, class; and the SVRZ and SIVRZ models include a resistant, R, subpopulation of cells with (partial or full) resistance to infection. The equations of the models are the following (for each model, we emphasise the added terms):

- SVZ

(2a) $$\frac{\mathrm{dS}}{\mathrm{dt}}=\overset{\text{growth}}{\overbrace{\mu .S}}-\overset{\text{mortality}}{\overbrace{d_{S}.S}}-\overset{\text{infection}}{\overbrace{\frac{\epsilon_{V}.\phi_{S}}{Q_{v}}.S.V}}-\overset{\text{grazing}}{\overbrace{g_{Z}.Z.S}}-\overset{\text{bottom}‐\mathrm{up}\;\text{control}}{\overbrace{\frac{S^{2}}{\tau_{K}.K}}}$$

(2b) $$\frac{\mathrm{dV}}{\mathrm{dt}}=\overset{\text{viral burst}}{\overbrace{\frac{\beta .\epsilon_{V}.\phi_{S}}{Q_{p}}.S.V}}-\overset{\text{viral decay}}{\overbrace{d_{V}.V}}-\overset{\text{viral adsorption}}{\overbrace{\frac{\phi_{S}}{Q_{p}}.S.V}}-\overset{\text{higher}‐\text{order}\;\text{losses}}{\overbrace{d_{V2}.V^{2}}}$$

(2c) $$\frac{\mathrm{dZ}}{\mathrm{dt}}=\overset{\text{grazing}}{\overbrace{\epsilon_{Z}.g_{Z}.S.Z}}-\overset{\text{mortality}}{\overbrace{d_{Z}.Z}}-\overset{\text{higher}‐\text{order}\;\text{losses}}{\overbrace{d_{Z2}.Z^{2}}}.$$

- SIVZ

(3a) $$\frac{\mathrm{dS}}{\mathrm{dt}}=\mu .S-d_{S}.S-\frac{\epsilon_{V}.\phi_{S}}{Q_{v}}.S.V-g_{Z}.Z.S-\frac{S^{2}}{\tau_{K}.K}$$

(3b) $$\frac{\mathrm{dI}}{\mathrm{dt}}=\overset{\text{infection}}{\overbrace{\frac{\epsilon_{V}.\phi_{S}}{Q_{v}}.S.V}}-\overset{\text{mortality}}{\overbrace{d_{S}.I}}-\overset{\text{lysis}}{\overbrace{\frac{1}{\tau }.I}}-\overset{\text{grazing}}{\overbrace{g_{Z}.Z.I}}$$

(3c) $$\frac{\mathrm{dV}}{\mathrm{dt}}=\overset{\text{viral burst}}{\overbrace{\frac{\beta }{\tau }.\frac{Q_{v}}{Q_{p}}.I}}-d_{V}.V-\frac{\phi_{S}}{Q_{p}}.S.V-d_{V2}.V^{2}$$

(3d) $$\frac{\mathrm{dZ}}{\mathrm{dt}}=\epsilon_{Z}.g_{Z}.\left(S+I\right).Z-d_{Z}.Z-d_{Z2}.Z^{2}.$$

- SVRZ

(4a) $$\frac{\mathrm{dS}}{\mathrm{dt}}=\mu .S-d_{S}.S-\frac{\epsilon_{V}.\phi_{S}}{Q_{v}}.S.V-g_{Z}.Z.S-\frac{S+R}{\tau_{K}.K}.S+\overset{\text{mutation}}{\overbrace{\mu .r.\left(\zeta .R-S\right)}}$$

(4b) $$\frac{\mathrm{dR}}{\mathrm{dt}}=\overset{\text{penalized growth}}{\overbrace{\mu .\zeta .R}}-d_{S}.R-\frac{S+R}{\tau_{K}.K}.R-\overset{\text{reduced infection}}{\overbrace{\frac{\epsilon_{\mathrm{VR}}.\phi_{R}}{Q_{v}}.R.V}}-g_{Z}.Z.R+\overset{\text{mutation}}{\overbrace{\mu .r.\left(S-\zeta .R\right)}}$$

(4c) $$\frac{\mathrm{dV}}{\mathrm{dt}}=\frac{\beta .\epsilon_{V}.\phi_{S}}{Q_{p}}.S.V+\frac{\beta .\epsilon_{\mathrm{VR}}.\phi_{R}}{Q_{p}}.R.V-d_{V}.V-\frac{\phi_{S}}{Q_{p}}.S.V-\frac{\phi_{R}}{Q_{p}}.S.V-d_{V2}.V^{2}$$

(4d) $$\frac{\mathrm{dZ}}{\mathrm{dt}}=\epsilon_{Z}.g_{Z}.\left(S+R\right).Z-d_{Z}.Z-d_{Z2}.Z^{2}.$$

- SIVRZ

(5a) $$\frac{\mathrm{dS}}{\mathrm{dt}}=\mu .S-d_{S}.S-\frac{S+R}{\tau_{K}.K}.S-\frac{\epsilon_{V}.\phi_{S}}{Q_{v}}.S.V-g_{Z}.Z.S+\mu .r.\left(\zeta .R-S\right)$$

(5b) $$\frac{\mathrm{dR}}{\mathrm{dt}}=\mu .\zeta .R-d_{S}.R-\frac{S+R}{\tau_{K}.K}.R-\frac{\epsilon_{\mathrm{VR}}.\phi_{R}}{Q_{v}}.R.V-g_{Z}.Z.R+\mu .r.\left(S-\zeta .R\right)$$

(5c) $$\frac{\mathrm{dI}}{\mathrm{dt}}=\frac{\epsilon_{V}.\phi_{S}}{Q_{v}}.S.V+\frac{\epsilon_{\mathrm{VR}}.\phi_{R}}{Q_{v}}.R.V-d_{S}.I-\frac{1}{\tau }.I-g_{Z}.Z.I$$

(5d) $$\frac{\mathrm{dV}}{\mathrm{dt}}=\frac{\beta }{\tau }.\frac{Q_{v}}{Q_{p}}.I-d_{V}.V-\frac{\phi_{S}}{Q_{p}}.S.V-\frac{\phi_{R}}{Q_{p}}.R.V-d_{V2}.V^{2}$$

(5e) $$\frac{\mathrm{dZ}}{\mathrm{dt}}=\epsilon_{Z}.g_{Z}.\left(S+I+R\right).Z-d_{Z}.Z-d_{Z2}.Z^{2}.$$

The SVZ model, used as a baseline model, corresponds to the simplest predator–prey model, which represents viral infection and lysis as a simple predator (Lotka 1920). In all models, we include the following:

- A carrying capacity term, K for the phytoplankton, to approximate bottom‐up control by nutrients which has relaxation time $\tau_{K}$ (see Section 2.6.1 for an approximation with the resource‐consumer model).
- Zooplankton grazing with a clearance rate $g_{Z}$, gross growth efficiency $\epsilon_{Z}$ and a linear per capita mortality rate of $d_{Z}$.
- The potential for inclusion of quadratic mortality terms for the virus ($d_{V2}$) and the zooplankton ($d_{Z2}$) (see Sections 2.5.2 and 2.5.5).

Each model either includes or does not include the I class (SIVZ and SIVRZ model) and/or a resistant type to the virus (R class, SVRZ and SIVRZ models). Models that include the I class introduce an additional parameter τ, the latent period of the virus, and a grazing term of the infected class by the zooplankton. This grazing pathway constitutes intraguild predation of the virus by the zooplankton, in the sense that zooplankton and viruses compete for the same phytoplankton host while zooplankton also consume virus‐infected cells (Holt and Polis 1997). Models that include the R class introduce a mutation term, r fraction, between the S and R class, extracellular resistance to the virus (through $\phi_{R}$) and intracellular resistance to the virus for the R type (through $\epsilon_{\mathrm{VR}}$). Extracellular resistance is modelled as a reduction in the adsorption rate while intracellular resistance is modelled as a decrease in the probability of an infection that results in the production of virus progeny. A cost of resistance ζ (fraction) is implemented as a reduction in maximum growth rate, consistent with generic resistance trade‐offs in host–virus systems (Våge et al. 2013). While costs of resistance are not universal, a moderate reduction (20%) was assumed as a plausible illustrative value, consistent with reported ranges in marine cyanobacteria (Lennon et al. 2007; Avrani et al. 2011). Note that to take into account the elemental quota of the zooplankton the grazing terms of the zooplankton (which we call $Z_{S\text{grazing}}$ and $Z_{I\text{grazing}}$ respectively for the S and I class) could be written as follows:

$Z_{S\text{grazing}}=-\frac{\phi_{Z}}{Q_{Z}}.S.Z$; $Z_{I\text{grazing}}=-\frac{\phi_{Z}}{Q_{Z}}.I.Z$ and $\frac{\mathrm{dZ}}{\mathrm{dt}}=\frac{\epsilon_{Z}^{\prime }.\phi_{Z}}{Q_{p}}.\left(S+I\right).Z+\ldots =\frac{\epsilon_{Z}.\phi_{Z}}{Q_{z}}.\left(S+I\right).Z+\ldots$.

Which gives: $g_{Z}=\frac{\phi_{Z}}{Q_{Z}}$ and $\epsilon_{Z}^{\prime }=\epsilon_{Z}.\frac{Q_{p}}{Q_{Z}}$. Where $\phi_{Z}$ is the zooplankton‐phytoplankton encounter rate, $Q_{z}$ is the zooplankton elemental quota and $\epsilon_{Z}^{\prime }$ is the grazing efficiency per prey cells consumed.

We summarise the default set of parameters associated with all models in Tables S1–S3.

### 2.3 Theoretical Equilibria, Stability and Alternate Stable States

For the SVZ model and the SIVZ model, five possible equilibria are possible:

- $S^{*},I^{*},V^{*},Z^{*}$
- $S^{*},0,0,Z^{*}$
- $S^{*},I^{*},V^{*},0$
- $S^{*},0,0,0$
- 0,0,0,0.

For the SVRZ and SIVRZ model, 11 possible equilibria are possible:

- $S^{*},I^{*},V^{*},R^{*},Z^{*}$
- $S^{*},I^{*},V^{*},\delta_{R^{*}},Z^{*}$
- $\delta_{S^{*}},I^{*},V^{*},R^{*},Z^{*}$
- $S^{*},I^{*},V^{*},\delta_{R^{*}},0$
- $S^{*},I^{*},V^{*},R^{*},0$
- $\delta_{S^{*}},I^{*},V^{*},R^{*},0$
- $S^{*},0,0,\delta_{R^{*}},Z^{*}$
- $\delta_{S^{*}},0,0,R^{*},Z^{*}$
- $S^{*},0,0,\delta_{R^{*}},0$
- $\delta_{S^{*}},0,0,R^{*},0$
- 0,0,0,0

Where $\delta_{X^{*}}$ is a state in which the variable X is in very low abundance: either S or R can be in such a state due to the mutation from one to another, while the other is abundant. Note that the five states where R is either 0 or $\delta_{R^{*}}$ can be mapped to the states of the SIVZ and SVZ models.

For the purpose of finding ecologically relevant equilibria, we derive the five possible equilibria of the SIVZ model (Equations (3a), (3b), (3c), (3d)) and 11 of the SIVRZ model (Equations (5a), (5b), (5c), (5d), (5e)) in two cases (Supporting Information):

- $d_{V2}=0$ and $d_{Z2}>0$
- $d_{V2}>0$ and $d_{Z2}>0$

We present the equilibria of the models including the I class—the equilibria of the other models can be calculated analogously and are included in the full model simulations.

### 2.4 Size and Elemental Quotas

In this study, we focus on four different types of phytoplankton: *Prochlorococcus*, *Synechococcus*, a picoeukaryote (non‐diatom), and a small diatom. Each phytoplankton type is considered separately with its own virus and grazer. The elemental concentration model we develop necessitates fixing their sizes to define their elemental quotas, that is, the amount of nitrogen in one phytoplankton cell, virion or grazer.

#### 2.4.1 Phytoplankton

For eukaryotes, we obtain nitrogen cell quotas following Menden‐Deuer and Lessard (2000) and the Redfield ratio (Redfield 1934):

- Diatoms:

(6) $$\log_{10}\left(Q_{\text{diatom}}\left(C\right)\right)=\left(-0.541+0.811.\log_{10}\left(\mathrm{Vol}\right)\right),$$

- Eukaryotic protists excluding diatoms:

(7) $$\log_{10}\left(Q_{\text{phytoeukaryotes}}\left(C\right)\right)=\left(-0.665+0.939.\log_{10}\left(\mathrm{Vol}\right)\right),$$

where Vol is the cell volume. We then convert to nitrogen units ($\text{μmolN}.{\text{cell}}^{-1}$):

(8) $$Q\left(N\right)=Q\left(C\right).10^{-6}.\frac{1}{\mathrm{MMC}}.\frac{16}{106},$$

where $\frac{16}{106}$ is the Redfield ratio of nitrogen to carbon and MMC is the molar mass of carbon.

For cyanobacteria, *Synechococcus* and *Prochlorococcus*, we use the carbon density of *Synechococcus* from Verity et al. (1992):

(9) $$Q_{\text{cyanobacteria}}\left(N\right)=d.\mathrm{Vol}.10^{-9}.\frac{1}{\mathrm{MMC}}.\frac{16}{106},$$

where d is the mass density of *Synechococcus*: *d* = 470 fgC.μm^−3^ (Verity et al. 1992) and consistent with other measurements for *Prochlorococcus* (Roth‐Rosenberg et al. 2021). For each phytoplankton type, we consider the smallest of each group as modelled in Dutkiewicz et al. (2020) (Table S1).

#### 2.4.2 Virus

For each class of phytoplankton, the size of the virus is set as the average size of viruses from the given group from the dataset of Edwards and Steward (2018) (Table S1). Empirical elemental quota relationships for viral capsids are set from Jover et al. (2014):

(10) $$Q_{\text{virus}}\left(N\right)=\frac{10^{6}}{N_{A}}\cdot \left(16{\left(r_{v}-2.5\right)}^{3}+36\left(7.5r_{v}^{2}-18.75r_{v}+15.63\right)\right)\cdot \mathrm{MMN},$$

where $r_{v}$ is the radius of the virion, $N_{A}$ is the Avogadro number and MMN is the molar mass of nitrogen. $Q_{\text{virus}}\left(N\right)$ is in $\text{μmolN}.{\text{virion}}^{-1}$.

#### 2.4.3 Zooplankton

We consider a grazer with a 2.5 μm radius for cyanobacteria and 5 μm for a picoeukaryote and a diatom. We follow Menden‐Deuer and Lessard (2000) for the size‐structured elemental concentrations:

(11) $$\log_{10}\left(Q_{\text{grazer}}\left(C\right)\right)=\left(-0.547+0.9.\log_{10}\left(\mathrm{Vol}\right)\right).$$

### 2.5 Life History Traits Models and Parameterisation

#### 2.5.1 Viral Burst Size and Latent Period

##### 2.5.1.1 Model Predictors

We used data from Edwards and Steward (2018) to calibrate models of burst size and latent period as a function of different host and virus traits. Burst size and latent period ($\log_{10}$) were found to be the most highly correlated with host volume ($\log_{10}$) and secondarily with the radius of the virus. In addition to these predictors, we add the virus type (ssDNA, dsDNA, ssRNA) and the host type (prokaryote, diatom and eukaryotes) as predictors. Pairs of host type‐virus type represented less than 5 times in the dataset are removed from the training set. For eukaryotes, we exclude classes of eukaryotes represented less than four times in the dataset (Pelagophyte, Chlorophyte, Raphidophyte and Cryptophyte), leaving three classes of non diatom eukaryotes: Prasinopyte, Haptophyte and Dinoflagellates. The same predictors were used for each type of model.

##### 2.5.1.2 Model Training

We used leave‐one‐out cross validation (typically used for small datasets) (Hastie et al. 2009) to assess the performance of four types of models: linear models, generalised additive models (GAM) (Wood 2004), random forest (RF) (Breiman and Cutler 2012), and single‐layer neural networks (NN) (Venables and Ripley 2002). The cross validation is also used to optimise the hyperparameters of the three latter models: the number of splines for GAM; the number of neurons, learning rate, and seed for NN; the number of trees and the number of variables to randomly sample as candidates at each split for RF. The NN model performed the best on average to minimise the root mean square error (RMSE) in leave‐one‐out cross validation (Figures S1–S4). The seed of the NN model for the latent period is not optimised as we empirically find that it overfits the dataset. The optimised NN models are used to set burst sizes and latent period (except for the burst size of diatom viruses, where we instead used the GAM model due to apparent overfitting of the NN model, Figures S1 and S2). Performances and fitting of the models are summarised for burst size in Figures S1 and S2 and latent period in Figures S3 and S4.

#### 2.5.2 Viral Quadratic Mortality

For the higher‐order quadratic mortality term of the virus, we use the value of Beckett et al. (2024), that fitted a quadratic mortality for *Prochlorococcus* viruses at station ALOHA (Table S2). The quadratic mortality term, $d_{V2}.V^{2}$, for the virus represents a generalised nonspecific loss term due, in part, to unspecific binding to other particles present in the ocean, including self collisions and adsorption to POM.

#### 2.5.3 Viral Encounter Rate

We use an encounter rate kernel that assumes that the swimming speed of the phytoplankton is considered to have a multiplicative effect on the diffusion kernel (Murray and Jackson 1992; Edwards et al. 2021):

(12) $$\phi_{\mathrm{th}}=\overset{\text{virus diffusion}}{\overbrace{4.\pi .\left(r_{\text{phyto}}+r_{\text{virus}}\right).D_{\text{virus}}}}.\overset{\text{phytoplankton swimming}}{\overbrace{\frac{1}{2}.\left(1+{\left(1+2.\frac{u_{\text{phyto}}.r_{\text{phyto}}}{D_{\text{virus}}}\right)}^{1/3}\right)}},$$

where $r_{\text{phyto}}$ is the radius of the phytoplankton. $u_{\text{phyto}}$ is the swimming speed of the phytoplankton (defined below). $D_{\text{virus}}$ is the diffusivity constant of the virus (defined below), the one of the phytoplankton being negligible in comparison. The diffusivity kernel of the virus is defined as follows (Einstein 1905):

(13) $$D_{\text{virus}}=\frac{k_{B}.T}{6.\pi .\eta .r_{\text{virus}}},$$

where $k_{B}$ is the Boltzman constant, T is the temperature in Kelvin, η is the dynamic viscosity of water. For non‐diatom eukaryote phytoplankton swimming speed we use the following empirical model (Kiørboe 2011; Talmy, Beckett, Taniguchi, et al. 2019):

(14) $$\log_{10}\left(u_{\text{phyto}}\right)=0.4+0.8.\log_{10}\left(2r_{\text{phyto}}\right),$$

where $r_{\text{phyto}}$ is in cm and $u_{\text{phyto}}$ in $\mathrm{cm}.s^{-1}$ (then converted in $m.s^{-1}$). For diatoms and cyanobacteria, we consider only diffusion (no swimming speed).

#### 2.5.4 Phytoplankton: Maximum Growth Rate and Half Saturation Constant

For the maximum growth rates (in $d^{-1}$), we follow the empirical allometric relationship with cell volume from Dutkiewicz et al. (2020):

(15) $$\mu_{\max}=a_{t}.{\mathrm{Vol}}^{b},$$

in which $a_{t}$ depends on the phytoplankton type (diatoms: 3.9, other eukaryotes: 1.4, cyanobacteria (*Prochlorococcus* and *Synechococcus*): 0.8). b is equal to −0.08 for diatoms and picoeukaryotes and 0.08 for cyanobacteria. For each of the four types (diatom, picoeukaryote, *Prochlorococcus* and *Synechococcus*), we consider a small cell of each type (Table S1). Each of them is infected by a virus of the average size of viruses from Edwards and Steward (2018) from the respective categories (Table S1). The volume of the phytoplankton and the radius of the virus also allows to set the burst size and latent period of each virus. For the nutrient dependency, $N_{c}$ the half saturation constant for population growth is set as followed:

(16) $$N_{c}=\frac{H_{c}.\mu_{\max}.Q_{\min}}{U_{\max}},$$

where $H_{c}$ is the cell nutrient uptake half saturation constant, controlling the efficiency of nutrient acquisition at the individual level, $Q_{\min}$ is the cell minimum stoichiometric quota, $U_{\max}$ is the maximal cell nutrient uptake rate. For all three parameters, an empirical allometric relationship with the cell volume Vol is used (see details in Dutkiewicz et al. 2020; Zakem et al. 2018). Finally, we assume non‐preferential grazing by zooplankton with respect to host infection or resistance status.

#### 2.5.5 Zooplankton Predation

We parameterise zooplankton predation following Dutkiewicz et al. (2020). We consider a zooplankton grazer with a grazing rate of 9.8 ${\left(\text{μmolN}.L^{-1}\right)}^{-1}.d^{-1}$, quadratic mortality of 1.4 ${\left(\text{μmolN}.L^{-1}\right)}^{-1}.d^{-1}$ and linear mortality of 0.067 $d^{-1}$ (Dutkiewicz et al. 2020; Dutkiewicz, Hickman, et al. 2015). Gross growth efficiency is set to 0.3 (Straile 1997). The quadratic mortality term, $d_{Z2}.Z^{2}$, represents higher order predation of the zooplankton which is not explicitly represented. The grazer of the cyanobacteria and eukaryotes (picoeukaryote (non‐diatom) and small diatom) have the same parameters except their size. Note that, unlike Dutkiewicz et al. (2020), we do not include a Hill‐type grazing formulation. We retain the same maximum grazing rate $g_{Z}$; rescaling it to account for the missing half‐saturation term did not improve model performance.

### 2.6 Simplified Epipelagic Environments

With the aim to better parameterise viral lysis models for large‐scale biogeochemical models, we construct idealised epipelagic environments that account for their carrying capacity (Equation 19), temperature and nutrient limitation of growth and mortality terms and biotic limitation of grazing.

#### 2.6.1 Approximation of the Resource‐Consumer Model by the Carrying Capacity Model

In biogeochemical models, limiting resources for phytoplankton growth, for example, nitrate, are explicitly represented. To simplify our model, we use a carrying capacity term instead. To make an equivalence between the two types of model we set the carrying capacity as the equilibrium concentration of the consumer in the resource‐consumer model with a constant nutrient concentration. The resource‐consumer model can be written as follow:

(17) $$\frac{\mathrm{dP}}{\mathrm{dt}}=\mu .\frac{N}{N+N_{c}}.P-d.P,$$

(18) $$\frac{\mathrm{dN}}{\mathrm{dt}}=-\mu .\frac{N}{N+N_{c}}.P+w.\left(N_{\text{deep}}-N\right),$$

where P is the phytoplankton concentration, N the nutrient concentration, $N_{c}$ is the half saturation constant, w is the surface‐deep mixing rate and $N_{\text{deep}}$ is the deep nutrient concentration, following Weitz et al. (2015). Assuming a constant nutrient concentration, we have:

(19) $$P^{*}=w.\left(N_{\text{deep}}-N\right).\frac{N+N_{c}}{\mu .N}.$$

$P^{*}$ is used as the carrying capacity K in the carrying capacity model (Table S3 for parameter values).

#### 2.6.2 Growth Limitations

We define two typical model epipelagic open ocean environments: oligotrophic and mesotrophic. To represent them in our models, in addition to modifying the carrying capacity, we add constant growth limitations:

- A constant penalty on the growth rate of the phytoplankton to represent changes in nutrient affinity considering a constant nutrient concentration. It is represented with a Michaelis–Menten term:
(20) $$\mu =\mu \left(N\right)=\mu .\frac{N}{N+N_{c}},$$
  where $N_{c}$ is the half saturation constant.
- A constant penalty/advantage on the growth rates and all mortality parameters through modulation by temperature following the Eppley curve, parameterised with values from Dutkiewicz et al. (2020):
(21) $$\gamma_{T}=\tau_{T}.e^{-A_{T}.\left(\frac{1}{T}-\frac{1}{T_{N}}\right)},$$
  where T is the temperature in Kelvin, $\tau_{T}$, $A_{T}$, $T_{N}$ are parameters regulating the maximum value and the shape of the limitation curve.

We summarise two sets of values chosen to be representative of the oligotrophic and mesopelagic environments respectively, as well as the effects of these terms in Table S3.

### 2.7 Coexistence Analysis

#### 2.7.1 Predator Growth Rates

To analyse the mechanisms facilitating coexistence in the SIVZ model, it is necessary to define the growth rates of the predators (Chesson 1982; Chesson and Ellner 1989; Ellner et al. 2019). The growth rate of the virus is set as the largest real part of the eigenvalues of the Jacobian J of the infected cell and virus, I,V subsystem (see Equations S49 and S50).

The per capita growth rate of the free virus is:

(22) $$\mu_{V}=\frac{\beta }{\tau }.\frac{Q_{v}}{Q_{p}}.\frac{I}{V}-d_{V}-\frac{\phi_{S}}{Q_{p}}.S-d_{V2}.V$$

The per capita growth rate of the zooplankton is:

(23) $$\mu_{Z}=\epsilon_{Z}.g_{Z}.\left(S+I\right)-d_{Z}-d_{Z2}.Z$$

#### 2.7.2 Modern Coexistence Theory

To assess the mechanisms of coexistence of the SIVZ model (without quadratic mortality terms of the predators), we perform an invasion analysis following Ellner et al. (2019). Briefly, Modern Coexistence Theory (Ellner et al. 2019) shows that nonlinear responses to fluctuations in resources—either exogenous or endogenous—can facilitate coexistence when species differ in how they respond to those fluctuations. We calculate the differences in growth rates between the invader species and the resident species in the case of both invasion (virus invading and zooplankton invading), and for the different cases of constant, varying and covarying resources (I class and S class, see Equation 12 from Ellner et al. (2019) and mathematical details including the definition of the *relative non linearity* in Supporting Information).

Coexistence is possible in the case of mutual invasibility, that is, if the invasion growth rate of each species invading the other is positive following Chesson's criterion (Chesson 1982; Chesson and Ellner 1989). For this analysis we consider the virus as the I,V subsystem (Equation S50). When fluctuation exists in the resident regime, we perform the analysis over one periodic cycle of the Susceptible type detected using the *find_peaks* function from the *SciPy* Python library (Virtanen et al. 2020). In the case of no fluctuation (e.g., resident Zooplankton), we perform the analysis over the last year of simulation (20 years).

#### 2.7.3 Growth Rate Analysis in the Coexistence Regime

To assess the effect of adding the I class on the growth rate of the virus and the zooplankton, we decompose the growth rate of the zooplankton and, in this case, of the free virus (Equation 22) in the coexistence regime, with or without fluctuations of their resources. The fluctuation free growth rate of predator P (either V or Z) defined as their respective growth rates when I and S concentrations are considered constant (Equation S53). The nonlinearity in S (respectively, in I) is quantified as the difference between the average growth rate under fluctuating S (or I) and the fluctuation‐free growth rate (Equations S54 and S55).

### 2.8 Fourier Analysis

To assess whether the system is stable or oscillatory outside of transient dynamics, we performed a Fourier analysis of the virus raw time series in the last year of simulation (of 20 years). The fft function from the *NumPy* Python library (Harris et al. 2020) is used and the frequency with the largest modulus is extracted. We consider the system to be oscillatory when the largest modulus is greater than 1.

### 2.9 Ecological Metrics

We define the percentage of infected cells as follows:

(24) $$\%I=100.\frac{I}{P},$$

where P=S+I+R is the total phytoplankton concentration. The percentage of resistant cells (without considering infected cells) is:

(25) $$\%R=100.\frac{R}{S+R}.$$

We define the per‐capita virus‐induced mortality, $m_{V}$, and zooplankton‐induced mortality, $m_{Z}$, as follows:

(26) $$m_{V}=\frac{I}{P}.\frac{1}{\tau },$$

(27) $$m_{Z}=g_{Z}.Z.$$

From these metrics, we define the percentage of virus‐induced mortality:

(28) $$\%V_{m}=100.\frac{m_{V}}{m_{V}+m_{Z}+d_{S}},$$

where $d_{S}$ is the cell mortality rate (natural cell death).

The net primary productivity at equilibrium is defined as the net photosynthesis rate of the phytoplankton (with contributions from susceptible and resistant cell classes):

(29) $$\mathrm{NPP}=\left(\mu .S+\mu .\zeta .R\right).\frac{106}{16},$$

where the C:N Redfield ratio $\frac{106}{16}$ is used to convert into units of $\text{μmolC}.d^{-1}.L^{-1}$.

### 2.10 Model Testing

#### 2.10.1 Target Concentrations

We define target concentrations based on the values of virus and phytoplankton concentration measured in the field, generally measured in $\text{individual}.L^{-1}$ (noted $\mathrm{ind}.L^{-1}$ for any tracer throughout the text) (Mojica et al. 2016; Leblanc et al. 2018; Carlson et al. 2022; Beckett et al. 2024) (including these targets in Table S4). For *Prochlorococcus*, the target concentration is defined as $1.5.10^{8}\;\mathrm{ind}.L^{-1}$ in an oligotrophic environment and $2.10^{8}\;\mathrm{ind}.L^{-1}$ in a model mesotrophic environment, while the target concentration of phages are, respectively, $5.10^{8}$ and $10^{9}\;\mathrm{ind}.L^{-1}$ (Mojica et al. 2016; Carlson et al. 2022). For picoeukaryotes (based on picoeukaryote from Mojica et al. 2016) and a small diatom (based on concentrations of small diatoms from Leblanc et al. 2018 and similar to concentration of nanoeukaryotes in Mojica et al. 2016), in a model mesotrophic environment, target concentrations are respectively $2.10^{7}$ and $2.10^{6}\;\mathrm{ind}.L^{-1}$ (Mojica et al. 2016; Leblanc et al. 2018) and $2.10^{7}$ and $10^{9}\;\mathrm{ind}.L^{-1}$ for their respective viruses. We set the same target concentration for the diatom virus as the concentration for the *Prochlorococcus* phage as the diatom virus is of a similar size and diatom viruses can reach very high concentrations in culture experiments (Tomaru et al. 2021; Arsenieff et al. 2019). Finally, measured values of $1–2.10^{5}\;\mathrm{ind}.L^{-1}$ for zooplankton are used (Schartau et al. 2010). We consider heterotrophic flagellates with a radius of 2.5 μm for cyanobacteria and a radius of 5 μm for the picoeukaryote and the small diatom (Schartau et al. 2010; Sherr and Sherr 1994). Note that our model does not include competition which occurs in the ocean. Therefore our target concentration reflect approximate expected values without competition. To further constrain the model outputs, we impose biologically realistic ranges for the percentage of infected cells (0.5%–10% in the model mesotrophic environment and 0%–5% in the model oligotrophic environment; Carlson et al. (2022)) and the proportion of virus‐induced mortality (0%–50%). For diatoms, the upper bound on the percentage of infected cells is relaxed due to limited empirical constraints. These bounds are not included in the target concentration error metric (see below), but they must be satisfied during the optimisation procedure (see Section 2.10.3).

#### 2.10.2 Model Error

To assess the error between target concentrations and modelled concentrations for an environment e, we define the total absolute error, in %, as the sum of absolute relative errors across tracers:

(30) $$D_{e}=\sum_{X=P,V,Z}\frac{|X_{s}^{e}-X_{t}^{e}|}{X_{t}^{e}},$$

where $X_{s}$ and $X_{t}$ are respectively the theoretical and targeted concentrations of a tracer (P, V or Z). We define the average absolute error across environments (O oligotrophic, M mesotrophic) as a weighted mean across environments:

(31) $$\bar{D}=\frac{1}{\sum_{e\in O,M}\alpha_{e}}\sum_{e\in O,M}\alpha_{e}.D_{e}.$$

For the picoeukaryote and the small diatom, a weight (parameter $\alpha_{e}$) of 2 (i.e., it is counted twice) is given to the error in the model mesotrophic environment as there exist no precise measurements of these organisms in oligotrophic environments (dominated by cyanobacteria) and target concentrations are more arbitrary.

#### 2.10.3 Parameter Optimisation

To minimise the error between theoretical model steady states and target concentrations we perform a grid search of the parameter space using theoretical equilibria of the SIVZ model (Supporting Information). The following parameters are searched: intracellular and extracellular resistance, the resistance cost, the linear and quadratic mortality of the virus (that can be null), and the quadratic mortality of the zooplankton. Explored ranges are summarised in Table 1. We define the extracellular resistance as the ratio between the theoretical encounter rate and the fitted adsorption rate ($\frac{\phi_{\mathrm{th}}}{\phi_{R}}$) while the intracellular resistance is defined as the inverse of the probability of infection ($\frac{1}{\epsilon_{\mathrm{VR}}}$). In our approach, the constraints on the percentage of infected cells and the percentage of virally‐induced mortality must be satisfied for an equilibrium to be valid.

### 2.11 Simulations and Criterion of Existence

Each model is simulated for 20 years using an integration time step of 30 min following the Runge–Kutta 4 integration scheme (Lotkin 1951) in a spatially implicit setup. Simulations are run for a range of parameters values for each model, where we choose to provide particular focus on variations in the adsorption rate. For the SIVZ and SIVRZ models, which include an infected cell class, we also include assessment with respect to varying the latent period. The burst size is fixed by the life history trait model (see Section 2.5). For the SIVRZ model we also explore the adsorption rate and the resistance strength space while the latent period and burst size are fixed by the life history traits models. To operationally assess the final state of the model, we check whether the concentration of each tracer, that is, each model compartment, was found to be greater than $1\;\mathrm{ind}.L^{-1}$ in the last year of simulation.

## 3 Results

### 3.1 Mechanisms of Coexistence Between the Virus and the Zooplankton

#### 3.1.1 The Inclusion of the Infected Class or the Resistant Type Facilitates Regimes of Coexistence

**FIGURE 3:**
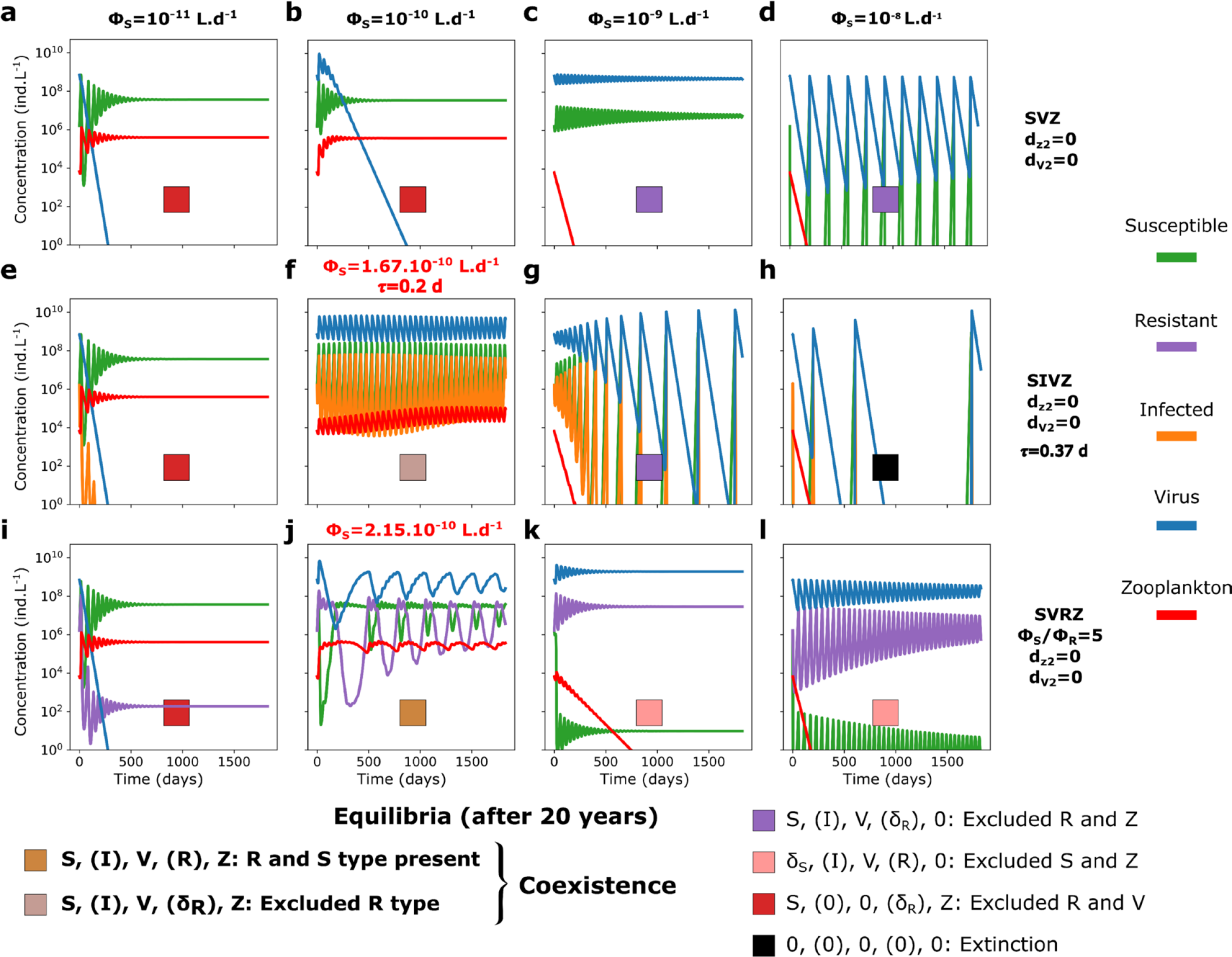
Five year time series of the SVZ, SIVZ and the SVRZ models for *Prochlorococcus* for four different adsorption rates of the virus without quadratic mortality terms. Adsorption rate from $\phi_{S}=10^{-11}\;L.d^{-1}$ to $\phi_{S}=10^{-8}\;L.d^{-1}$ ($\epsilon_{V}=1$) for (a–d) the SVZ model, (e–h) the SIVZ model and (i–l) the SVRZ model. The tracers' molar concentrations were converted to count concentrations using the cellular and virion quotas (see Figure S5 for time series in molar concentrations). A latent period of 0.37days and a burst size of 15 were used as parameterized by the life history trait model (see Section 2). To showscase the coexistence regime facilitated by the I class (Infected) and R (Resistant), we slightly changed the parameters (f) τ=0.2d and $\phi_{S}=1.67.10^{-10}\;L.d^{-1}$ and (j) $\phi_{S}=2.15.10^{-10}\;L.d^{-1}$ (highlighted in red). For these values of adsorption rate, the zooplankton is excluded in the SVZ model (Figure 4a).

We begin by assessing how choice of model structural assumptions promotes coexistence between a virus and a zooplankton population both exploiting the same *Prochlorococcus* population (used as a model organism). We compare simulations across a range of viral adsorption rates for the baseline competition model (SVZ, Figure 2a) and for the more complex SIVZ and SVRZ models (Figure 2b,c), which incorporate, respectively, an infected host class I and a resistant host type R. Ecologically, increasing viral adsorption rate increases the probability that phytoplankton losses occur through viral lysis rather than grazing, thereby altering the balance between the two predators. For the SVZ model (Figure 2a; Equations (2a), (2b), (2c)), which resembles a Lotka–Volterra system with one prey and two predators (Lotka 1920), the virus is excluded for low values of the adsorption rate of the virus (Figure 3a,b) while the zooplankton is excluded for higher values (Figure 3c,d). This aligns with the competitive exclusion principle (Armstrong and McGehee 1980), which states that two species competing for the same resource with linear growth rates cannot coexist indefinitely.

**FIGURE 4:**
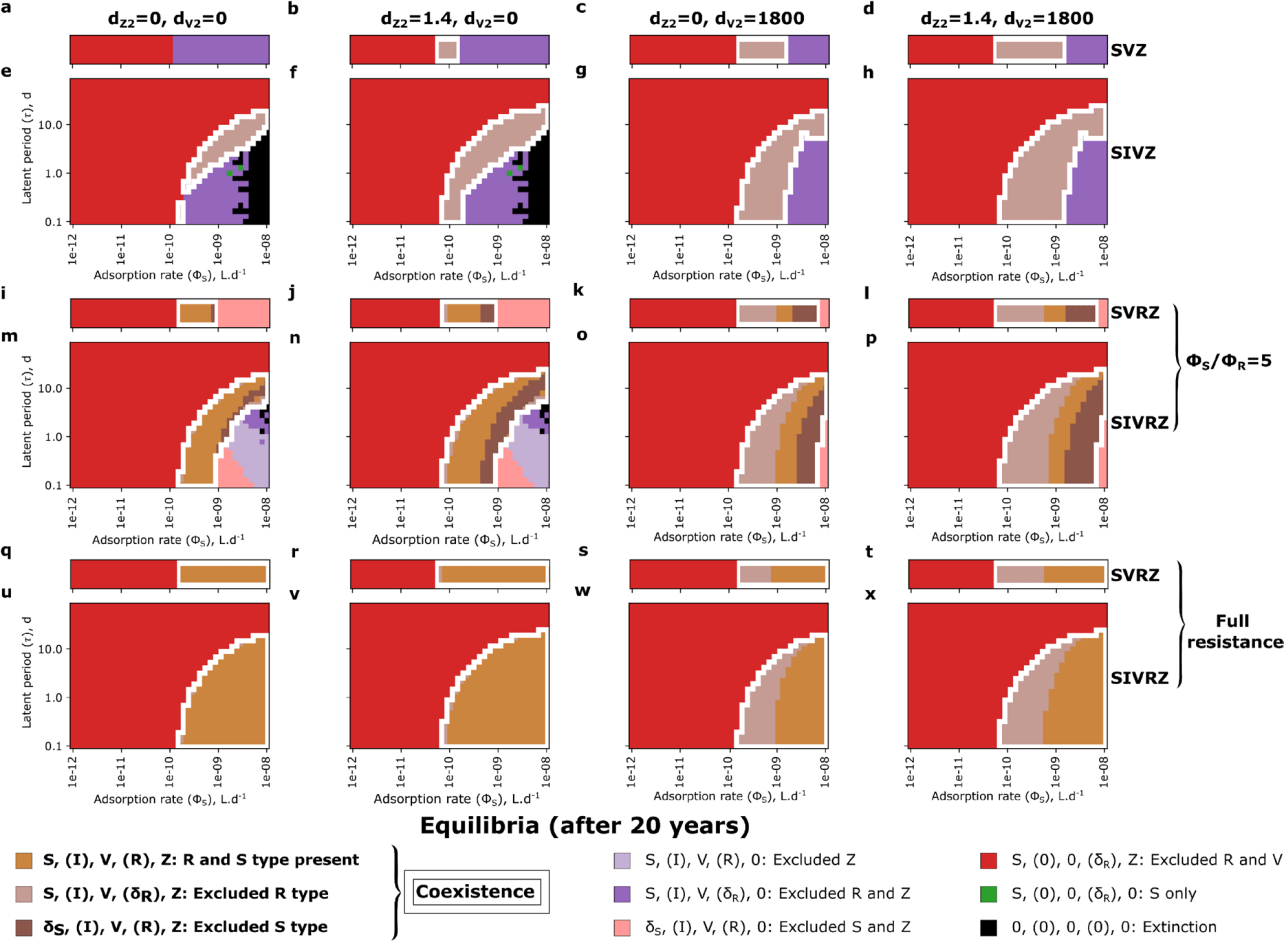
Approximate simulated equilibrium regimes of a virus and a zooplankton modelled as predators of a *Prochlorococcus* for different types of viral lysis models across the adsorption rate and the latent period parameter space. (a–d) SVZ model. (e–h) SIVZ model. (i–l) Extracellular SVRZ model with a partially resistant type: $\frac{\phi_{S}}{\phi_{R}}=5$. (m–p) Extracellular SIVRZ model with a partially resistant type: $\frac{\phi_{S}}{\phi_{R}}=5$. (q–t) Extracellular SVRZ model with a fully resistant type. (u–x) Extracellular SIVRZ model with a fully resistant type. From left to right panels: Quadratic mortality terms respectively absent, present for the zooplankton only, present for the virus only, and present for both the zooplankton and the virus. The quadratic mortality terms are in $\left(\text{μmolN}.L^{-1}\right).d^{-1}$. The SVZ model was run for 25 years to allow full exclusion while the other models were run for 20 years. In the latter case, the equilibrium might not be reached yet. In addition some numerical instabilities might explain the fuzzy transitions between regions with different regimes. To define the equilibrium reached in each simulation we test whether each tracer had a concentration superior to 1 $\mathrm{ind}.L^{-1}$ in the last year of simulation. For each panel, the white contour denotes the coexistence regime.

To investigate model complexification that may facilitate coexistence between the two predators, we first introduce an infected class of phytoplankton (I): the SIVZ model (Figure 1b; Equations (3a), (3b), (3c), (3d)). Introducing the infected class to the model adds a new parameter: the latent period of infection that we fix to 0.37d using the life history trait model (Section 2, Figure S3 and Table S1). This is representative of measured *Prochlorococcus* cyanophage infections from Mruwat et al. (2021) and Carlson et al. (2022). For the lowest adsorption value, the virus is still excluded (Figure 3e). However, we find that by shortening the latent period and increasing the next adsorption rate tested (to $1.167\times 10^{-10}\;L.d^{-1}$), an oscillatory coexistence regime results (Figure 3f). For this value of adsorption rate, the zooplankton is excluded from the SVZ model (Figure 4a). When further increasing the adsorption rate of the virus, the zooplankton is either excluded (Figure 3g) or the system collapses, that is, all tracers are excluded (Figure 3h). Notably, for the highest adsorption rate, the latent period generates larger oscillations in the system leading to system collapse, which was not the case for the SVZ model.

An additional model complexification that may facilitate the coexistence of the virus and the zooplankton is the introduction of a resistant type (R) with an associated cost to its growth rate. This is represented by the SVRZ model (Figure 2c, Equations (4a), (4b), (4c), (4d)). We specifically consider extracellular resistance to viral infection that prevent adsorption, thus the resistance is modelled by a reduced viral adsorption rate, denoted by the parameter $\phi_{R}$ (Equations 4b and 4c). We arbitrarily set a 5‐fold ratio between the adsorption rate of the susceptible cell population and the one of the population of resistant cells. A slight increase in the adsorption rate of the virus from $10^{-10}$ to $2.15.10^{-10}\;L.d^{-1}$, results in a coexistence regime between the virus and the zooplankton with oscillatory behaviour of all classes (Figure 3j). For higher adsorption rate values, the zooplankton is excluded. In this regime, the resistant phytoplankton type largely dominates in abundance relative to the susceptible. The latter is only sustained in very low abundance via mutation (Figure 3k,l).

Together, these results show that viral adsorption rate governs how host mortality is partitioned among grazing, infection and resistance pathways, thereby determining virus–zooplankton coexistence. In the baseline SVZ model, increasing adsorption shifts mortality from grazing to viral lysis, leading to competitive exclusion. By contrast, introducing an infected class or a resistant host type redistributes viral‐induced mortality across host compartments, generating coexistence regimes with oscillatory dynamics. The following sections examine the coexistence mechanisms underlying these regimes and their interaction in the combined SIVRZ model.

#### 3.1.2 A Trade‐Off Between Latent Period and Virulence Facilitates Coexistence

To analyse coexistence regimes beyond single parameter dynamics, we compute coexistence regimes across the virulence spectrum of the virus. For simplicity, we refer to the viral adsorption rate ($\phi_{S}$) multiplied by the probability of infection resulting in the production of new virions ($\epsilon_{V}$) as its virulence throughout the remainder of the text. Operationally, we use the terms very, moderate or mildly virulent to denote effective adsorption rates of $\epsilon_{V}.\phi_{S}>10^{-9}$, $10^{-10}<\epsilon_{V}.\phi_{S}<10^{-9}$ and $\epsilon_{V}.\phi_{S}<10^{-10}\;L.d^{-1}$ respectively. For simplicity, we set $\epsilon_{V}=1$ and vary virulence only through $\phi_{S}$; $\epsilon_{V}$ is allowed to vary in the last section focused on parameter fitting. For the SVZ model of *Prochlorococcus*, as expected from previously described time series, coexistence occurs between the phytoplankton and either the virus or the zooplankton for all adsorption rates tested, but not both simultaneously. The outcome depends on the viral adsorption rate, with one predator excluding the other (Figure 4a).

For the SIVZ model, we explore a parameter range for the latent period beyond measured maximum latent periods (maximum of 1.75 days in Edwards and Steward 2018) and most likely beyond what is expected in the ocean, to assess the effect of this parameter across a large range and visualise transitions in model outputs. We find that the inclusion of the I class alone, regardless of whether it is grazed or not can facilitate coexistence between the virus and zooplankton. Virus‐grazer coexistence is observed only when the carrying capacity term for the phytoplankton is retained and occurs within a narrow range of the viral latent period and adsorption rate (Figure 4e). Only moderate to very virulent viruses are able to coexist with the zooplankton in the region of the virulence spectrum where the virus excludes the grazer in the SVZ model (Figure 4a vs. Figure 4e). As the virulence increases, the latent period required for the virus to persist also increases, reaching up to approximately 10 days for the most virulent viruses (much longer than the doubling time of the host, Figure 4e). These coexistence regimes are found to be unstable, generating limit cycles that are away from the equilibrium points of the system (Figure S6). This suggests a trade‐off between the viral adsorption rate and latent period to facilitate coexistence, where sufficiently small oscillations are necessary to prevent the system from collapsing. The inclusion of the I class appears to have two opposing effects. First, in the case of coexistence, the time delay between the viral adsorption to the host and the burst of new viruses generates a limit cycle, with a short time shift between the peaks (highs and lows) of the virus and zooplankton population dynamics (Figure S6). These peaks may either follow one another in the same order (Figure S6a) or not (Figure S6b). Second, for very virulent viruses with a relatively short latent period, the time delay causes strong oscillations in the SIV dynamics (within the SIVZ model), which can drive the system to extinction (Figure 4e), a behaviour not seen in the SVZ model (Figure 4a). Interestingly, the inclusion of the I class can either support coexistence or lead to extinction, depending on the underlying ecological life‐history traits.

#### 3.1.3 Modern Coexistence Theory of the SIVZ Model: Non Linear Oscillations of the Susceptible Type Facilitates Coexistence

**FIGURE 5:**
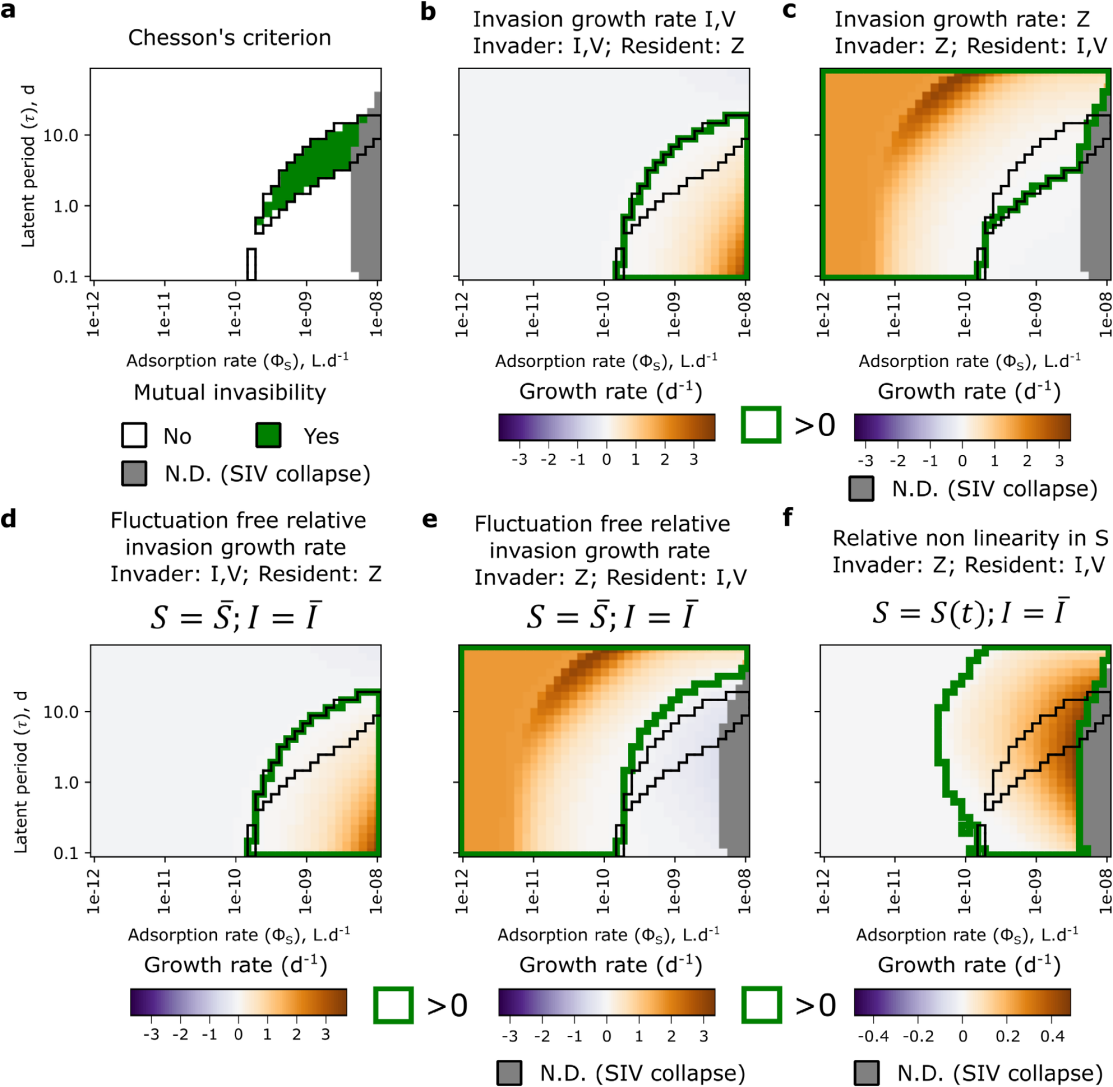
Modern coexistence theory (MCT) analysis of the mechanisms facilitating coexistence of the zooplankton and the virus in the SIVZ model without quadratic mortality of the predators. (a) Chesson's criterion of mutual invasibility across 10 replicates of the Modern Coexistence theory analysis: In the green area, mutual invasion, that is, both the invasion growth rate of the virus and the zooplankton are positive for all replicates of the MCT analysis. In the grey area, the analysis is not possible as the SIV model collapses in this region. (b) Invasion growth rate of the virus (considered as the subsystem IV). (c) Invasion growth rate of the zooplankton. (d) Fluctuation free relative growth rate of the virus when the virus is invading. (e) Fluctuation free relative growth rate of the zooplankton when the zooplankton is invading. (f) Relative nonlinearity in S when the zooplankton is invading. For each panel, the coexistence regime reached by the SIVZ model after 20 years of simulations, without quadratic mortality terms for the predators, is represented as the black contour. N.D., not defined.

To further investigate how the inclusion of the infected class (I) facilitates the coexistence regime, we performed an invasibility analysis following Chesson's mutual invasibility criterion of coexistence (Chesson 1982; Chesson and Ellner 1989). When the resident state exhibits fluctuations, invasibility analysis is performed over a single periodic cycle of S (Section 2). Across 10 replicates of the invasibility analysis, we find good agreement between mutual invasibility and the coexistence regime (Figure 5a,b). However, the region of coexistence extends slightly beyond the parameter range where Chesson's criterion holds. For highly virulent viruses with long latent periods, the SIV model collapses, while the SIVZ model still supports coexistence, suggesting a stabilising effect of the zooplankton in this case. The sensitivity of Chesson's criterion was 0.76 when requiring all invasibility analysis replicates to be valid and 0.91 when requiring at least one replicate to be valid; these values decreased to 0.71 and 0.85, respectively, when including the SIV collapse region as false negatives. It is to be noted that some dynamics within the coexistence regime are still on a slow trajectory towards exclusion, as simulations over 20 years might not fully capture long‐term transients. Visualising the invasion growth rate of the virus (Figure 5b) and of the zooplankton (Figure 5c) shows that the coexistence regime exactly matches the upper border of positive virus invasion growth rate while it approximately corresponds to the lower border of positive invasion growth rate of the zooplankton (likely due to numerical instabilities in the invasion analysis).

Following Modern Coexistence Theory (MCT, Ellner et al. 2019), we further decompose these invasion growth rates into their relative components (Equation 26). First, when the virus invades the SZ system, the fluctuation‐free and total invasion growth rates coincide (Figure 5b,d), as SZ dynamics always converge to a stable node equilibrium (no fluctuations). In contrast, when the zooplankton invades the resident SIV system, the fluctuation free relative invasion growth rate of the zooplankton is negative in the coexistence regime (Figure 5e). Nevertheless, this is compensated by a positive relative nonlinearity in S (Figure 5f): the zooplankton takes better advantages of the fluctuations of S than does the virus. This suggests that this nonlinearity acts as the main mechanism of coexistence between the virus and the zooplankton. Importantly, this nonlinearity alone is not sufficient for coexistence, as it is positive over a broad region of the parameter space. For coexistence to be enabled, in addition to a positive invasion growth rate of the virus, the positive relative nonlinearity in S must be strong enough to offset the negative fluctuation‐free invasion growth rate when the zooplankton invades.

To further characterise the underlying mechanism underpinning the relative nonlinearity in S, we decompose the average growth rates of the free virus and the zooplankton in the coexistence regime (SIVZ model) with or without fluctuations of the I and S classes (Section 2, Equations (28), (29), (30)). We find that the fluctuation‐free growth rate of the free virus, that is, considering the concentration of the I and S class to be constant (set as their mean over a period cycle of S), varies from near zero to extremely high values ($>10^{3}\;d^{-1}$, Figure S7a). As expected from the formula of the free virus growth rate (Equation 25), this result comes from a negative linear component in I for the growth rate of the free virus (Figure S7b). The inclusion of the infected class thus has a detrimental effect on the growth rate of the free virus, which in turn facilitates the coexistence. The lower clearance rate ($g_{Z}$) of the zooplankton compared to the infection rate of the virus ($\frac{\epsilon_{V}.\phi_{S}}{Q_{v}}$, which is the viral clearance rate in the SVZ model) is effectively compensated by a reduced growth rate of the free virus population due to the dynamics of the oscillating infected population. In turn, this reduced growth rate of the free virus allows the zooplankton to take better advantage of the susceptible cells through the relative nonlinearity in S described in the MCT analysis. For the zooplankton, as expected from the linear growth rate of the latter, we observe no significant nonlinearities in the coexistence regime.

#### 3.1.4 The Inclusion of Resistance Facilitates Coexistence Through Resource Partitioning

We assess the effects of inclusion of a resistant phytoplankton type alongside the susceptible phytoplankter, the SVRZ model. We examine two scenarios: one where the resistant type exhibits partial resistance with a ratio of $\frac{\phi_{S}}{\phi_{R}}=5$, and another where the resistance is complete, meaning $\phi_{R}=0$ (fully resistant type). For the partially resistant type, a coexistence regime is observed in the region characterised by viruses of moderate virulence (Figure 4i). In contrast, for the fully resistant type, this region extends into the domain characterised by very virulent viruses (Figure 4q). As anticipated, these coexistence regions are located within the part of the virulence spectrum where the virus excludes the grazer in the SVZ model (Figure 4a vs. Figure 4i,q). Intuitively, the introduction of a resistant type decreases the viral growth rate, while allowing the zooplankton to graze on the resistant type, which experiences reduced susceptibility to viral effects. To illustrate this, we plot the dynamics of the SVRZ model within the coexistence regime (Figure S8). Two distinct dynamic patterns emerge in this regime: either slowly dampened oscillations or sustained periodic oscillations. For a moderately virulent virus, the oscillations persist after 20 years of simulations, with the virus and the resistant type oscillating in opposite phases (Figure S8a,c). In the case of a slightly more virulent virus and a partially resistant type, the oscillations of the resistant type and the zooplankton dampen more quickly, while those of the susceptible type and the virus remain coupled (Figure S8b). Finally, for moderately virulent viruses, in the case of full resistance, we observe a stable equilibrium (Figure S8d). Importantly, adding the resistant type reduces the total virus abundance, and as viral virulence increases, the system transitions from dominance of the susceptible cell type to the resistant cell type. Overall, these results confirm that introducing a resistant type creates resource partitioning between the virus and the zooplankton, with the virus mainly targeting the susceptible type (though depending on the relative abundances of the S and R type), while the zooplankton consumes both types, primarily feeding on the more abundant one.

To further investigate this model, we set the latent period at 0.37days (≈9 h, based on the life history trait model) and examine the impact of resistance strength with a constant cost on the growth rate of the R type (Figure S9). As the virus virulence increases, the resistance strength required to achieve the coexistence state also increases (Figure S9a). Adding the infected class (SIVRZ model, Figure 1f, Equations (5a), (5b), (5c), (5d), (5e)) the state where the zooplankton is excluded in the SVRZ model now supports coexistence between the S and R types (Figure S9a vs. S9e). Finally, we also test for an intracellular resistance model by modulating the probability of entering the infected state (parameter $\epsilon_{\mathrm{VR}}$). In this case, the coexistence state is reached at lower resistance strengths for very virulent viruses (Figure S9a,e vs. S9i,m). These findings suggest that extracellular resistance might be more favourable to invade a population of susceptible cells and become dominant.

#### 3.1.5 The Inclusion of Higher‐Order Mortality Terms for Predators Facilitates Coexistence and Stabilisation

We next examine how density‐dependent predator losses expand the parameter space facilitating coexistence between viruses and zooplankton and stabilise population dynamics. Quadratic mortality terms are not introduced to limit predator biomass per se, but to assess how the form of predator losses reshapes coexistence in systems where multiple predators exploit the same phytoplankton population. In ocean ecosystem models, higher‐order mortality terms represented by quadratic mortality terms are often used for grazers as a closure term for the system (e.g., Dutkiewicz et al. 2020) and have also been used as a loss term for viruses (Talmy, Beckett, Zhang, et al. 2019; Beckett et al. 2024). For the zooplankton, the quadratic mortality represents top‐down control by higher predators, assumed to scale with zooplankton density. For the virus, the quadratic mortality represents losses due to unspecific binding, for example, collision with particulate matter. We find that the inclusion of a quadratic mortality term (Steele 1974) for the zooplankton in the SVZ model facilitates the coexistence regime in the adsorption rate region where the virus was previously excluded (Figure 4b vs. 4a). Similarly, the inclusion of a quadratic mortality term for the virus facilitates coexistence in the region where the zooplankton was previously excluded (Figure 4c vs. 4a). The inclusion of both terms results in both regions entering the coexistence regime (Figure 4d). In the SIVZ, SVRZ and SIVRZ models, the effects are similar (Figures 4 and S9). Notably, for very virulent viruses and a partially resistant type ($\frac{\phi_{S}}{\mathrm{\phi R}}=5$), the inclusion of these higher‐order loss terms also facilitates a coexistence state (of V and Z) where the R type dominates, excluding the S type (dark brown regions, Figure 4j–l,n–p). Interestingly, the region of R type dominance is larger for extracellular resistance than for intracellular resistance. This further suggests that extracellular resistance might be more beneficial assuming the same cost of resistance (Figure S9d,h vs. S9l,p). Additionally, in the $S\left(I\right)\mathrm{VRZ}$ models, the inclusion of the virus quadratic mortality extends the region where the S type dominates, excluding the R type (light brown region, Figure 4k,l,o,p,s,t,w,x). For the $S\left(I\right)\mathrm{VRZ}$ model with a partially resistant type, as the virulence of the virus increases, there is a succession of coexistence states: first, the S type dominates (light brown region), then the S and R types coexist (gold brown region), and finally, the R type dominates (dark brown region, Figure 4l,p). It should be noted that the cases of S and R type dominance correspond to the same equilibrium. The only difference is the cost to the growth rate for the latter. These results suggest that higher‐order losses favour phenotype selection by promoting coexistence regimes dominated either by the susceptible or the partially resistant type.

It is well known that quadratic mortality terms have a stabilising effect on community dynamics (Poethke and Kirchberg 1987). To verify this in our system, we derive approximate model equilibria for the SIVZ and SIVRZ models in two cases: $d_{Z2}>0$ with either $d_{V2}=0$ or $d_{V2}>0$ (see Supporting Information for the mathematical derivations, Equations S14–S48, and Figures S10–S14). We simulate the dynamics of the models and calculate the approximate theory (Section 2) for four types of small phytoplankton: a small *Prochlorococcus*, a small *Synechococcus*, a picoeukaryote (non‐diatom), and a small diatom (Table S1). We find a strong agreement between the approximate theory and the simulations (SIVZ, Figure S10 and SIVRZ, Figure S12), including system stability (shown in Figures S11 and S13), when compared to the Fourier analysis (Figure S14). The quadratic mortality terms strongly stabilise the system, with the viral quadratic mortality term acting to stabilise viral population dynamics for more virulent viruses. Oscillatory dynamics are still maintained only for very virulent viruses and long latent periods (Figure S14). As expected from encounter rate theory (Huisman and Weissing 1999; Levin et al. 1977; Talmy, Beckett, Zhang, et al. 2019), the coexistence regime shifts towards more virulent viruses for larger organisms (Figures S10 and S12). The approximate theory is less accurate for larger organisms (diatoms), particularly for very virulent viruses in the SIVZ model (Figure S10p) and for long latent periods in the SIVRZ model, but it still effectively captures the coexistence regime (Figure S12p). Overall, these results underscore the stabilising influence and broader coexistence regimes that emerge when higher‐order loss processes, formulated as quadratic mortality terms, are included in biogeochemical models.

Together, these results show that higher‐order loss terms widen and reshape coexistence regimes, while competitive exclusion persists in some regions of the parameter space. These density‐dependent losses stabilise community dynamics and promote phenotype selection.

### 3.2 Towards the Integration of Viral Lysis in Ocean Ecosystem Models

#### 3.2.1 The Effect of Including a Virus: Critical Transitions and Impact on the System

**FIGURE 6:**
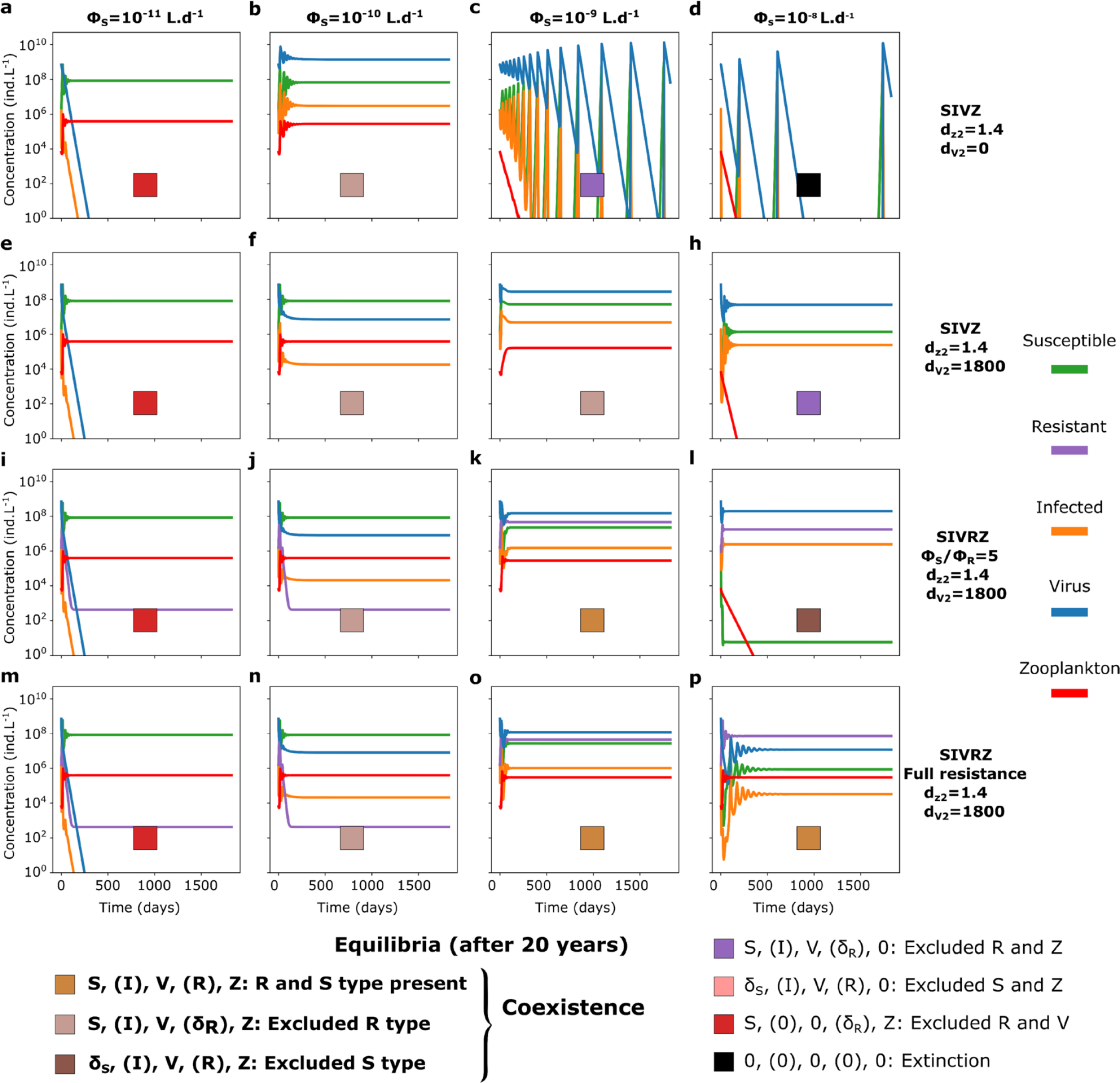
Model output of 5 year time series of the SIVZ and the extracellular SIVRZ models for *Prochlorococcus* for four different adsorption rates of the virus and different resistant type strength. Adsorption rate from $\phi_{S}=10^{-11}\;L.d^{-1}$ to $\phi_{S}=10^{-8}\;L.d^{-1}$ ($\epsilon_{V}=1$) for the SIVZ model (a–d) without and (e–h) with the quadratic mortality term of the virus, for the SIVRZ model (i–l) with a partially resistant type and (m–p) a fully resistant type, both with the quadratic mortality term of the virus. The tracers' molar concentrations were converted in count concentrations using the cellular and virion quotas (see Figure S13 for time series in molar concentrations). A latent period of 0.37days and a burst size of 15 were used as parameterized by the life history trait model (see Section 2). The quadratic mortality terms are in ${\left(\text{μmolN}.L^{-1}\right)}^{-1}.d^{-1}$.

A crucial challenge in implementing viral lysis models in ocean ecosystem models is in assessing the viral impact on the ecosystem and identifying ecological parameter ranges that produce realistic concentrations of different biogeochemical tracers. To address this issue, we first analyse the impact of including the virus on system dynamics and equilibria for the set of simulations including quadratic mortality terms (generally used in large scale ecosystem models) (Figure 6 for count concentrations and Figure S15 for nitrogen molar concentrations). We use a fixed latent period for our model *Prochlorococcus*, as determined by the life history trait model (Figures S3 and S4, Table S1). For all models we consider, mildly virulent viruses are excluded after approximately 1 year of simulation (Figure 6a,e,i,m). As the virulence of the virus increases, stable coexistence equilibria are first enabled by the addition of zooplankton quadratic mortality (Figure 6b), but these equilibria become unstable for more virulent viruses (Figure 6d,e). High virus count concentrations are reached in this case, generally much higher than the phytoplankton count concentration. Adding a viral quadratic mortality term strongly decreases the equilibrium virus concentration below the phytoplankton concentration (Figure 6f). As virulence increases, the virus concentration increases, leading to a decrease in phytoplankton concentration (Figure 6g). In the cases of very virulent viruses, virus concentration decreases and is accompanied by a sharp decline in phytoplankton concentration (Figure 6h). In the case of a partially resistant type, the same behaviour is observed (Figure 6j,k), except for very virulent viruses, where the regime allows a new virus concentration peak where the resistant type dominates but the zooplankton is excluded (Figure 6l). For the fully resistant type (Figure 6m–p) coexistence with the zooplankton is maintained for very virulent viruses (Figure 6p).

**FIGURE 7:**
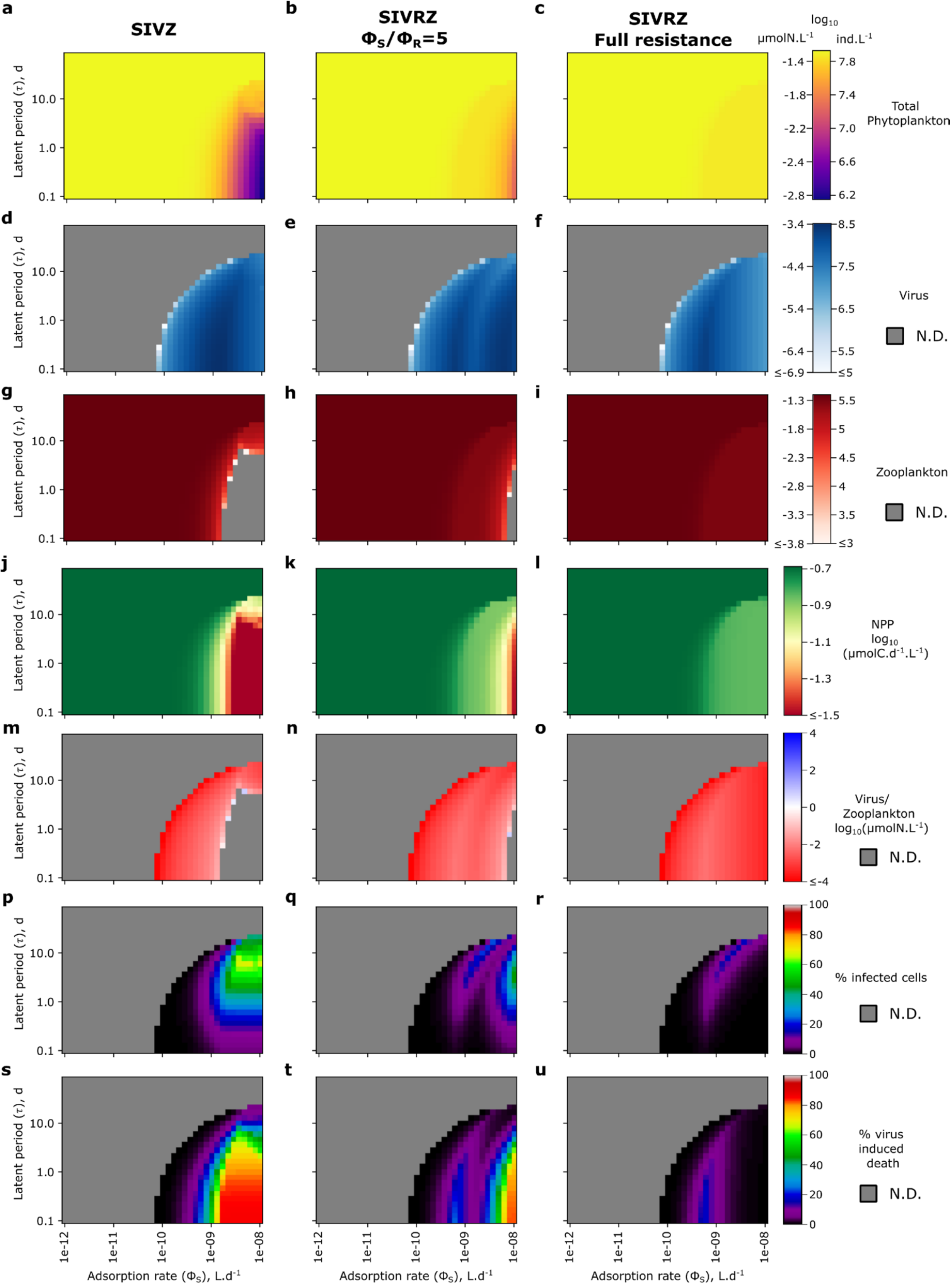
Equilibrium concentration and ecological features of the SIVZ and the extracellular resistance SIVRZ models for *Prochlorococcus* across the adsorption rate and latent period parameter space. From left to right panels: SIVZ model, SIVRZ model with a partially resistant type: $\frac{\phi_{S}}{\phi_{R}}=10$, and SIVRZ model with a fully resistant type. (a–c) Total phytoplankton concentration. (d–f) Virus concentration. (g–i) Zooplankton concentration. (j–l) Ratio of virus to zooplankton concentrations. (m–o) Percentage of infected cells. (p–r) Percentage of virus induced death. (s‐u) percentage of virus induced mortality. In panels (m–o), the grey areas fall outside the coexistence domain, so these metrics are not defined. N.D., not defined: The tracer is excluded or one of the necessary tracers to define the metric is excluded.

To better understand these effects, we visualise the equilibrium concentrations of total phytoplankton, virus and zooplankton (averaged over the last year in the case of oscillations) across the virulence spectrum and the latent period parameter space for the different models in the set of simulations that include the quadratic mortality terms (Figure 7a–o). The total concentration of phytoplankton is significantly decreased by the presence of the virus only when the virus becomes sufficiently virulent, its encounter rate (infection rate) surpassing that of the zooplankton (Figure 7a). The total phytoplankton concentration decreases from $∼10^{-1.2}$ to $10^{-3.1}\;\text{μmolN}.L^{-1}$ ($∼10^{8}$ to $10^{6}\;\mathrm{ind}.L^{-1}$), slightly less than two orders of magnitude, for the most virulent viruses. Including a partially resistant cell type reduces this effect (decreasing to $10^{-2}\;\text{μmolN}.L^{-1}$, i.e., $10^{7.25}\;\mathrm{ind}.L^{-1}$) and the transitions between the three coexistence regimes (Figure 4p) become apparent (Figure 4b). In the case of the fully resistant type, the R type dominated regime is not reached (Figure 7c). The decomposition of the phytoplankton population into susceptible, infected and resistant types, along with the percentage of resistant cells (relative to S+R), shows a rapid transition from a susceptible‐dominated population to a resistant‐dominated population as virulence increases (Figure S16g–p). For the zooplankton, as the virulence of the virus increases, its equilibrium concentration is similarly affected (Figure 7g–i, decreasing from $∼10^{-1.3}$ to $10^{-2.3}\;\text{μmolN}.L^{-1}$, i.e., $∼10^{5.5}$ to $10^{4.5}\;\mathrm{ind}.L^{-1}$) and is excluded for the most virulent virus in the SIVRZ model (Figure 7g). For the virus, a relatively broad concentration peak is observed for moderately virulent viruses in the SIVZ model (Figure 7e), which narrows in the SIVRZ model as the resistant type takes over (Figure 7e,f). For the SIVRZ model with a partially resistant type a second peak of virus concentration is observed corresponding to the regime of R dominance (Figure 7e). Conversely, in the case of the fully resistant type, the coexistence of the R and S types for very virulent viruses is enabled (full coexistence regime, Figure 4x). Surprisingly, in this case, the equilibrium viral concentration becomes independent of the latent period and decreases relative to viral concentrations in the S and R types dominated regimes (Figure 7e,f and the theoretical equilibria, Equations S42 and S43). The quadratic mortality term used for the virus appears to be relatively high (Beckett et al. 2024), so the maximum concentrations reached are around $∼10^{-3.4}\;\text{μmolN}.L^{-1}$, i.e., $∼10^{8.5}\;\mathrm{ind}.L^{-1}$. Overall, extending the latent period (τ) shifts the transitions between states towards more virulent viruses, with a modest effect for τ<1day and a more pronounced effect as the latent period is extended. Extending τ also narrows the zone of maximum viral concentration. We also estimate the impact of the virus on Net Primary Productivity (NPP, Figure 7j–l) a key metric for ocean ecosystems. NPP is directly related to phytoplankton concentration (Equation 29, Figure 7a–c). Consequently, very virulent viruses have a dramatic effect that decreases NPP (Figure 7j). Although the decrease in NPP is partially mitigated by the inclusion of the resistant type, the resistance cost decreases NPP when comparing the S type dominant regime to the mixed or R type dominant regimes (Figure 7k,l).

We next investigate the relative roles of the two predators in the system, along with the percentage of infections induced by the virus at equilibrium (Figure 7m–u). The zooplankton is generally more abundant in molar concentration than the virus, with a minimal molar ratio occurring at the maximum of free virus concentration (Figure 7m–o). For short latent periods (τ<1day), the percentage of infected cells is generally low, transitioning from near 0%–10% in the SIVZ model as virulence increases (Figure 7p). For the SIVRZ model, the highest infection percentages are found around the virus concentration peaks, although they remain quite low (<10%) when the latent period is sufficiently short (Figure 7p–r). Extending the latent period generally increases importantly the percentages of infected cells at equilibrium which also increases with the viral adsorption rate although to a lesser extent (Figure 7p–r). The percentage of virus‐induced mortality increases drastically with the virulence of the virus. For the SIVZ model, it transitions from nearly 0% to ∼90% over ∼1.3 orders of magnitude in the virulence spectrum (Figure 7s), highlighting the strong sensitivity of the system to the virulence of the virus. This effect is primarily driven by the decrease in abundance of the zooplankton and does not necessarily imply a much higher percentage of infected cells (for short latent periods such as the one of cyanophages). Finally, adding the partially resistant type creates two peaks in the maximal percentage of virus‐induced mortality (Figure 7t), while the fully resistant type results in a single peak (Figure 7u). These two peaks align with the two peaks of viral concentration corresponding to the regimes of S and R dominance respectively.

#### 3.2.2 Bridging Model and Field Ecology: Models Suggest the Dominance of Viral Resistance

**FIGURE 8:**
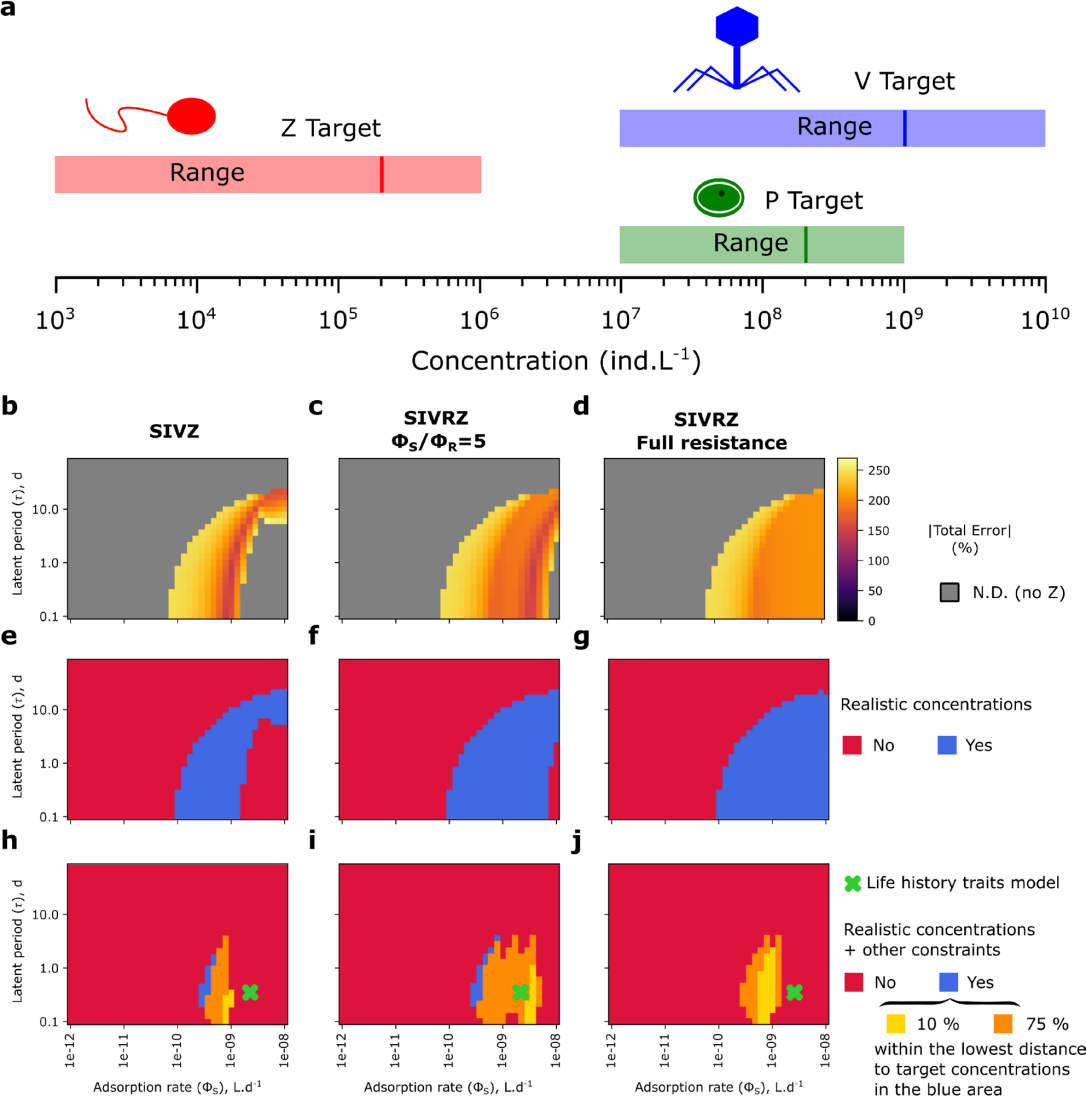
Model output compared to expected concentration and ecological features measured in the field across the adsorption rate and latent period parameter space. (a) Target and concentration ranges in a mesotrophic environment for the zooplankton (red), *Prochlorococcus* (green) and the virus (blue). (b–d) From left to right panels: SIVZ model, SIVRZ model with a partially resistant type: $\frac{\phi_{S}}{\phi_{R}}=10$, SIVRZ model with a fully resistant type: (b–d) Distance to target concentrations defined in Table S4, (e–g) Realistic concentrations in the range defined in Table S4 and (h–j) Realistic concentrations and constraints on other expected ecological features (percentage of infected cells, percentage of virus‐induced mortality and V/P ratio, see Table S4). The green cross represents the life history traits model. N.D., not defined: At least one tracer is excluded.

We assess the performance of each model against marine field measurements to identify parameter combinations that yield potentially realistic ecological outcomes. We define concentration ranges and targets for total phytoplankton, viruses and zooplankton, together with expected ranges for the percentage of infected cells and virus‐induced mortality in two idealised epipelagic environments (Table S4; Mojica et al. 2016; Carlson et al. 2022; Schartau et al. 2010; Section 2; Figure 8a). To illustrate the in situ measurements for the oligotrophic environment, we show the distributions from Carlson et al. (2022) in the NPSG (Figure S17, latitude < 33° N). The measured concentrations in the literature were reported in $\text{cell}.L^{-1}$ and $\text{virion}.L^{-1}$ so we convert to this unit for this analysis. Using the set of default parameters (Table S2), the SIVZ model minimises the distance to target concentrations in the model mesotrophic environment for moderately virulent viruses near the maximum concentration of free virus (Figure 8b). Decomposing this distance among the different tracers shows that it is minimised around the peak concentration of free virus for all tracers, except for the zooplankton in the SIVRZ model with the fully resistant type (Figure S18). For the SIVRZ model with a partially resistant type, the minimum distance to the target concentrations is found in the regime dominated by the resistant type (Figure 8c). Conversely, the model with a fully resistant type shows poorer performance (Figure 8d). When the target concentration assumptions are removed, the entire coexistence regime falls within realistic concentration ranges for all models (Figure 8e–g). However, further constraining the output with the percentage of infected cells, virus‐induced mortality, and a V/P count ratio greater than 1 (Figure S19), a much narrower region of the virulence spectrum and latent period parameter space aligns with observed empirical measurements (Figure 8h–j). These regions rarely encompass the coexistence regime including both the S and R types in abundance; instead, they mainly occur in regions where only one type is dominant (Figure 4 vs. 8h–j).

Importantly, the ‘realistic’ ecological parameter space is found within a virulence range with a lower adsorption rate, compared to a theoretical encounter model by Murray and Jackson (1992), where the swimming speed of the phytoplankton (zero in the case of cyanobacteria and diatoms) is considered to have a multiplicative effect on diffusion (Figure 8h–j, Equation 13, Section 2). The SIVRZ model with partial resistance represents the best match, as it minimises the error to target concentrations close to the life‐history trait model while also satisfying the other ecological constraints that we impose (Figure 8i). In general, these results suggest that including a level of extracellular resistance (compared to the encounter rate model) to the virus leads to a more accurate representation of the equilibrium achieved (Figure 8i).

To further test this hypothesis, we leverage the theoretical equilibria of the SIVZ model (Supporting Information). We allow for resistance and cost on the maximal growth rate, to minimise model error in modelling target concentrations for both a mesotrophic and an oligotrophic environment (avoiding overfitting). The procedure is followed for four types of phytoplankton: *Prochlorococcus*, *Synechococcus*, a picoeukaryote (non‐diatom) and a small diatom (Table S4). The theoretical equilibria allow us to exhaustively explore a large ecological parameter space using a grid search approach. We choose to focus on the intracellular and extracellular resistance, the resistance cost, the linear and quadratic mortality of the virus (that can be set to equal zero) and the quadratic mortality of the zooplankton (Table 1). We define the extracellular resistance as the ratio between the theoretical encounter rate and the fitted adsorption rate ($\frac{\phi_{\mathrm{th}}}{\phi_{R}}$) while the intracellular resistance is defined as the inverse of the probability of infection where new virions are produced ($\frac{1}{\epsilon_{\mathrm{VR}}}$). We find that the optimal extracellular resistance is around ∼3−20 for *Prochlorococcus*, *Synechococcus* and the picoeukaryote, while it is ∼270 for a small diatom. Intracellular resistance factors were found to be in the same order of magnitude as extracellular resistance. Multiplying both types of resistance, we get total resistance factors of 46, 29, 5 and 272 respectively for *Prochlorococcus*, *Synechococcus*, the picoeukaryote and the small diatom in order to minimise distance to target concentrations. Among the 200 best‐fitting models, extracellular and intracellular resistance were significantly anticorrelated (p<0.0001) for *Prochlorococcus*, *Synechococcus* and the small diatom, with correlation coefficients of r=−0.58, −0.71 and −0.53 respectively. The correlation was weaker for the eukaryote (r=−0.22, p<0.01), but the best‐fitting models exhibited a narrow range of extracellular resistance values. A resistance cost (1−ζ) of ∼0−0.5 is found to be a good fit across organisms although other parameters might also contribute to decreasing the effective growth rate in larger ocean models. The linear and quadratic mortality rates of the virus in best‐fit models are lower than the default parameters, notably the best‐fit linear mortality rates are consistent with laboratory‐based decay measurements, on the order of 0.01–0.03 $d^{-1}$ (Maidanik et al. 2022). The quadratic mortality of the virus appeared relatively poorly constrained across the 200 best‐fitting models for the four phytoplankton types. Finally, the best‐fitting models suggest a higher quadratic mortality for the zooplankton compared to the default value used although the fitted range of the 200 best‐fitting models remained wide and it might depend on the grazing function used.

We typically find low fitting errors in the differences between target and the modelled concentrations of the best fitting models (Table 2). Target virus concentrations are generally achieved for all four organisms. Generally, the percentages of infected cells reported by the models are small for cyanobacteria (0.75%–2.0% in the oligotrophic environment) and 3.1% for the picoeukaryote, which are in good agreement compared to average oligotrophic field measurements for cyanobacteria (Carlson et al. 2022; Beckett et al. 2024 and Figure S17). In the mesotrophic environment, the percentage of infected cells were 1.4% and 4.1% for *Prochlorococcus* and *Synechococcus* respectively which is below peak percentages observed in such environments (Carlson et al. 2022). The observed peaks most likely come from out of equilibrium dynamics or increase in latent period which our model does not allow here. Our fitted models also correspond to a relatively low percentage of virus‐induced mortality between 7.4% (*Prochlorococcus*) and 17% (*Synechococcus*) in agreement with oligotrophic field measurements (Mruwat et al. 2021; Beckett et al. 2024). In the mesotrophic environment, these percentages of virus‐induced mortality increase to a range of ∼12% (*Prochlorococcus*) to ∼38% (picoeukaryote). For the small diatom, the percentages of infected cells reaches ∼8% (most likely due to the longer latent period) in the mesotrophic environment but still corresponds to a relatively low percentage of virus‐induced mortality (15%).

**TABLE 1:** Parameter optimisation to target concentrations for an oligotrophic and a mesotrophic simplified epipelagic environment using the approximate theoretical equilibria for the four phytoplankton types.

Variable		*Prochlorococcus*	*Synechococcus*	Picoeukaryote	Diatom
Adsorption rate $\phi_{R}$ $10^{-12}-10^{-8}$	Best	$1.7.10^{-10}$	$2.15.10^{-10}$	$1.7.10^{-9}$	$1.3.10^{-10}$
	Range	$1.6.10^{-11}-7.7.10^{-10}$	$7.7.10^{-11}-1.7.10^{-9}$	$7.7.10^{-10}-1.7.10^{-9}$	$7.7.10^{-11}-10^{-8}$
Extracellular resistance $\frac{\phi_{\mathrm{th}}}{\phi_{R}}$	Best	12.9	17.6	3.3	271.6
	Range	3.6−129	2.3−48.8	3.3−7	3.5−453
Intracellular resistance $\frac{1}{\epsilon_{\mathrm{VR}}}$ $1-10^{4}$	Best	3.6	1.7	1.7	1
	Range	1−7.7	1−12.9	1−21.5	1−60
Total resistance $\frac{\phi_{\mathrm{th}}}{\phi_{R}}.\frac{1}{\epsilon_{\mathrm{VR}}}$	Best	46	29	5	272
	Range	10−129	13−63	3−70	126−453
Resistance cost 1−ζ 0−0.5	Best	0.05	0	0	0.4
	Range	0−0.5	0−0.3	0−0.2	0.2−0.5
Linear V mortality $d_{V}$ 0.01−1	Best	0.01	0.01	0.01	0.01
	Range	0.01−0.1	0.01−0.05	0.01−0.5	0.01
Quadratic V mortality $d_{V2}$ 0−2000	Best	70	90	1200	100
	Range	20−500	30−200	80−2000	70−400
Quadratic Z mortality $d_{Z2}$ 2−30	Best	12	12	22	14
	Range	10−14	10−18	12−30	10−30

*Note:* The best parameter value is defined as the one minimising the average absolute total error across the two environments for a given phytoplankton. The parameter range reflects the minimum and maximum value of a parameter within the first 200 best‐fitting models. Parameter ranges tested are indicated below each parameter. Adsorption rate is in $L.d^{-1}$, extracellular, intracellular resistance and resistance cost are unitless, linear mortality is in $d^{-1}$ and quadratic mortality terms are in ${\left(\text{μmolN}.L^{-1}\right)}^{-1}.d^{-1}$. Parameters that are kept fixed are described in Table S1–S3.

**TABLE 2:** Model performances compared to target concentrations for a simplified oligotrophic and mesotrophic epipelagic environment for the four types of phytoplankton.

Ocean		*Prochlorococcus*	*Synechococcus*	Picoeukaryote	Diatom
Oligotrophic	Minimum total error (%)	9	24	36	78
	Error range (%)	5−41	8−64	14−128	12−158
	Tot P	$1.4.10^{8}\;\left(-6.2\%\right)$	$2.3.10^{7}\;\left(+13.6\%\right)$	$1.1.10^{7}\;\left(+9.2\%\right)$	$5.5.10^{5}\;\left(+10.6\%\right)$
	V	$5.08.10^{8}\;\left(+1.7\%\right)$	$4.9.10^{8}\;\left(-2.5\%\right)$	$9.4.10^{7}\;\left(-5.7\%\right)$	$1.7.10^{8}\;\left(-14.3\%\right)$
	Z	$1.01.10^{5}\;\left(+1.2\%\right)$	$1.1.10^{5}\;\left(+7.5\%\right)$	$1.2.10^{4}\;\left(+21\%\right)$	$1.5.10^{4}\;\left(+53.4\%\right)$
	% Inf	0.75	2.05	3.1	1.7
	% V kill	7.4	17.4	27.6	6.5
	V/P	3.6	21.3	8.5	310
Mesotrophic	Minimum total error (%)	21	29	53	28
	Error range (%)	4−44	6−64	13−70	16−88
	Tot P	$2.1.10^{8}\;\left(+4.8\%\right)$	$3.7.10^{7}\;\left(-25\%\right)$	$2.10^{7}\;\left(-0.8\%\right)$	$1.5.10^{6}\;\left(-25.0\%\right)$
	V	$10^{9}\;\left(-0.3\%\right)$	$1.10^{9}\;\left(+2.5\%\right)$	$2.10^{8}\;\left(+1.4\%\right)$	$1.10^{9}\;\left(+2.9\%\right)$
	Z	$1.7.10^{5}\;\left(-15.7\%\right)$	$2.10^{5}\left(-0.4\%\right)$	$2.4.10^{4}\left(-51.0\%\right)$	$4.8.10^{4}\;\left(-0.3\%\right)$
	% Inf	1.4	4.10	6.3	7.9
	% V kill	12.0	25.7	38.4	15.1
	V/P	4.8	27	10	690
Weighted average	Minimum error (%)	15	26	47	45
	Error Range (%)	15−24	26−37	47−52	45−64

*Note:* The absolute percentage error (rows Best and Range) represents the total error across target environments cumulating errors to the target concentrations of the phytoplankton, the zooplankton and the virus. For each environment, the ‘minimum error’ corresponds to the error of best model for which the average absolute error across environments is minimised. The ‘Range’ reflects the minimum and maximum values of the first 100 best models. For TotP, V and Z, the equilibrium value is indicated in $\mathrm{ind}.L^{-1}$ and the minimum percentage error to the target is in parenthesis. For % Inf, % V Kill and V/P the equilibrium value is shown.

## 4 Discussion

We proposed several models that incorporate viral lysis of phytoplankton designed for integration into large‐scale ocean ecosystem models. Our approach transforms the SIV count model (Levin et al. 1977; Weitz 2015) into an elemental concentration SIV model that incorporates cellular and virion quota (Jover et al. 2014). We explicitly wrote elemental fluxes equations linked to the viral ‘shunt’ (Fuhrman 1999; Wilhelm and Suttle 1999) and the viral ‘shuttle’ (Sullivan et al. 2017; Guidi et al. 2015; Zimmerman et al. 2020). This step is essential for integration into large‐scale biogeochemical models, as it accounts for the distinct stoichiometry of viruses and their hosts, particularly their C:N:P ratios (Redfield 1934; Jover et al. 2014) which is expected to have consequences on DOM and POM stoichiometric composition. To validate the feasibility of integrating viral lysis models into large‐scale biogeochemical frameworks, we explored the mechanisms that could facilitate the coexistence of both a virus and a zooplankton preying on the same phytoplankton. Inclusion of the infected class (I) had a dual effect, either enabling limit cycles or driving the system to extinction through oscillatory dynamics. These oscillations in the infected class may play a crucial role in spatially resolved models by inducing out‐of‐equilibrium conditions, particularly in transition zones (Carlson et al. 2022). Next, the addition of a resistant type facilitated the coexistence of the two predators through niche partitioning. In particular, for the most virulent viruses, full resistance enabled the coexistence of both susceptible and resistant types, while partial resistance led to the exclusion of the susceptible type. The latter case (partial extracellular resistance) corresponds to the coexistence regime of a less virulent virus and includes an eventual cost to the growth rate of the phytoplankton. The inclusion of this kind of resistant type in biogeochemical models, although it may be computationally costly, could lead to the system alternating between different coexistence regimes, with a potential decrease of primary productivity due to the resistance cost. The resistance cost may depend on the mutation (Bohannan and Lenski 2000; Chao et al. 1977) and follow trade‐offs (Våge et al. 2013). In addition, the range of resistance, that is, the resistance to multiple viruses, which we do not resolve here, can vary (Avrani and Lindell 2015). Importantly, natural populations might evolve to a reduced resistance range and favour maximal growth rates through compensatory mutations (Avrani and Lindell 2015). Finally, the inclusion of higher‐order quadratic mortality terms for the predators expanded the coexistence regimes by stabilising the system. Quadratic mortality terms also have a controlling role on the concentrations reached by the predators. These terms are not well constrained and here we fitted values that are lower than those previously reported for viruses (Beckett et al. 2024) and higher than those used for zooplankton (Dutkiewicz, Morris, et al. 2015). Importantly, in large scale ecosystem models, other mechanisms of coexistence might be at play like advection by currents and the size fractionation of zooplankton grazers (Dutkiewicz et al. 2020).

We evaluated the impact of introducing the virus into the system, focusing on its effects on phytoplankton mortality and abundance, as well as the partitioning between zooplankton and virus‐induced mortality. The outcomes were strongly influenced by the virulence of the virus: mild viruses had minimal impact, whereas highly virulent viruses induced substantial changes in the system's dynamics and equilibrium. As previously reported, a maximal concentration of free viruses was observed for mildly virulent strains, whereas the most virulent viruses were less abundant, consistent with field observations of cyanophages infecting cyanobacteria populations in the global oceans (Maidanik et al. 2022). In addition, the system exhibited strong sensitivity, characterised by steep transitions in its ecological properties. In particular, we observed rapid shifts in the percentage of infected cells and virus‐induced mortality across the virulence spectrum. The percentage of infected cells was predominantly influenced by the latent period, while virus‐induced mortality showed a pronounced increase with higher viral adsorption rates. Thus, a high percentage of infected cells does not necessarily correspond to a high percentage of virus‐induced mortality, and vice versa. A highly virulent virus with a short latent period, despite its low abundance, can drive substantial phytoplankton mortality even when the percentage of infected cells at equilibrium is low. In the model, this phenomenon is driven primarily by the partial exclusion of zooplankton, which may, for instance, explain the negative correlations between viral and grazing rates observed in field studies (Biggs et al. 2021). These results further suggest that transient dynamics could readily emerge in spatially explicit models, potentially explaining the variability in ecological properties observed in the field, such as the percentage of infected cells and virus‐induced mortality (Mojica et al. 2016; Biggs et al. 2021; Carlson et al. 2022; Beckett et al. 2024).

Finally, we compared the outputs of our models to field measurements of virus, phytoplankton and zooplankton concentrations. Using the default ecological parameter set, the SIVZ and SIVRZ models performed similarly in minimising the distance to target concentrations. However, the SIVRZ model aligned more closely with the life history trait model describing the encounter rate between phytoplankton and their viruses. Notably and as expected (Weitz et al. 2015), this result suggests that incorporating inefficiencies and/or resistance into the encounter rate model provides a better fit to empirical measurements. Interestingly, modulating both extracellular resistance, directly affecting the adsorption rate and intracellular resistance, that decreased the probability of infection leading to the production of new virions, were necessary to fit the models to field measurements with the relative, total resistance factor ranging from low to medium to high, for picoeukaryotes, cyanobacteria and diatoms, respectively. Nevertheless, a certain degree of uncertainty remains in this parameterisation. Anti‐phage intracellular defence mechanisms are well‐documented (Labrie et al. 2010; Doron et al. 2018; Rousset et al. 2022), although some remain unidentified in marine cyanobacteria (Zborowsky and Lindell 2019). Notably, studies have shown that cyanophage resistance is predominantly extracellular against specialist phages (Zborowsky and Lindell 2019), which target the most abundant high‐light–adapted *Prochlorococcus* strains (Sullivan et al. 2003; Dekel‐Bird et al. 2015). In contrast, resistance is primarily intracellular against generalist phages (Zborowsky and Lindell 2019), which infect the less abundant *Synechococcus* and low‐light–adapted *Prochlorococcus* ecotypes (Sullivan et al. 2003; Dekel‐Bird et al. 2015). These observations support our findings, suggesting that both types of resistance are important in marine phytoplankton. Future models may wish to include the potential for intracellular resistance to halt infections at various stages (Zborowsky and Lindell 2019). In our approach, we assumed that intracellular resistance reduced the probability of transitioning to the infected state that successfully lead to the production of new virions. This could be further explored by modelling a transition rate from the infected class back to the susceptible class or by using more complex models that include intermediate infected states (Hinson et al. 2023; Dominguez‐Mirazo et al. 2024).

Importantly, the encounter rate and the adsorption rate can be influenced by several factors not explored in our models. The encounter rate represents the biophysical maximum rate of interaction, while the adsorption rate reflects the actual rate of successful attachment, which is a fraction of the encounter rate. For instance, not all virions attach to their host upon encounter, as receptor heterogeneity on the cell surface (Murray and Jackson 1992; Talmy, Beckett, Taniguchi, et al. 2019) and cell surface properties (Yamada et al. 2023) can limit attachment. Furthermore, turbulent diffusion can either increase or decrease encounter rates depending on the turbulence intensity, with high turbulence potentially reducing encounter rates (Stocker 2012; Pecseli et al. 2014). Additionally, the adsorption rate might be decreased due to nonspecific binding to other particles in the environment, such as cellular debris or other cells (Yamada et al. 2020), as well as through the formation of marine snow via the viral ‘shuttle’ mechanism (Sullivan et al. 2017; Guidi et al. 2015; Zimmerman et al. 2020). This effect could be represented in a model as a Hill function inhibiting viral adsorption to the host (Igler 2022). The magnitude of this effect may vary significantly in large‐scale ocean models and in field conditions due to the pronounced heterogeneity of marine environments. These effects may not be as pronounced as the extracellular resistance proposed here, especially in oligotrophic and mesotrophic environments, such as those modelled here, which have low POM concentrations (Tanioka et al. 2022) but high dissolved organic matter (DOM) concentrations (Hansell and Orellana 2021). The extracellular resistance used here can thus be viewed as the cumulative effect of these abiotic mechanisms in addition to biological resistance. Nevertheless, further research is needed to accurately account for and parameterise these effects.

Our community ecology model comes with caveats. First, despite identifying potential flux terms, we did not explicitly account for feedback with the microbial loop. This would require the explicit inclusion of heterotrophic bacteria and inorganic nutrients in the model (Weitz et al. 2015). Heterotrophic bacteria remineralise DON to dissolved inorganic nitrogen (DIN), thereby increasing nutrient availability to phototrophs, which is expected, in turn, to increase primary productivity (Fuhrman 1999; Wilhelm and Suttle 1999; Weitz et al. 2015). Nonetheless, the system of ordinary differential equations we propose (Equations (1a), (1b), (1c)) and the fluxes to DON and PON (Equations 1d and 1e) could be sufficient for integration of viral lysis into large‐scale ocean ecosystem models. To fully close the model, one should also redistribute mortality terms to the DIN, DON and PON compartments. Future work will seek to couple models such as those we develop here into more complex ecosystem models.

Despite its relative simplicity, the SIVRZ model, with a dominant R type, can effectively recapitulate the observed ecology of viral infections in oligotrophic and mesotrophic environments. By integrating algebraic theoretical results, field measurements and life history trait models, we are able to accurately reproduce viral and phytoplankton concentrations, percentages of infected cells, and virus‐induced mortality in both prokaryotic and eukaryotic phytoplankton. We characterised the effect of adding an infected class, which can have a dual impact on system stability, either stabilising or destabilizing, depending on the trade‐off between the latent period and the adsorption rate of the virus. In addition, the inclusion of the I class enables the model to accurately recapitulate the percentages of infected cells observed in the field. The percentage of infected cells in our model does not necessarily reflect the percentage of virus‐induced mortality, as it depends on the virulence of the virus and whether it impacts the zooplankton. Finally, our models suggest that incorporating significant extracellular resistance into biophysical encounter rate models may be critical for accurately modelling viral lysis in large biogeochemical models, which is consistent with the measured resistance of phytoplankton strains Here we developed only one host‐one virus models and did not account for viral (Gregory et al. 2019) and phytoplankton diversity (Dutkiewicz et al. 2020). The inclusion of multiple host‐virus pairs is expected to increase phytoplankton coexistence (Thingstad 2000; Avrani et al. 2011, 2012; Flynn et al. 2022). However, in systems with multiple host‐virus pairs it might be challenging to maintain coexistence of all virus‐host pairs (i.e., certain virus might be excluded and not others). We also used a very generic model of lytic viral infection and neglected the potential uptake of virions by grazers (DeLong et al. 2022). Other types of viral infection cycles exist, such as persistent infections (Tuttle and Buchan 2020; Sanchez‐Martinez et al. 2025) or chronic infections, for instance in *G. huxleyi*, where virions are continuously produced by exocytosis (Homola et al. 2024).

Integrating viral lysis in ocean ecosystem models may improve estimates of carbon cycling in the oceans and quantify the relative role of viruses and zooplankton in phytoplankton mortality. The inclusion of viruses might also reduce the magnitude of discrepancies between models shown to be linked to the parameterisation of grazing (Rohr et al. 2023). In closing, we anticipate that the inclusion of viral lysis into large ecosystem models will identify knowledge gaps and catalyse experimental and field‐based studies to assess how viruses shape community ecology and biogeochemical cycles.

## Author Contributions

Conceptualisation: P.F., S.J.B., D.D., E.C., C.L.F., D.L., D.T., S.D., J.S.W. Methodology: P.F. Investigation: P.F. Model development: P.F., S.J.B., D.D., E.C., C.L.F., D.L., D.T., S.D., J.S.W. Code review: E.C. Writing – original draft: P.F. Writing – review and editing: P.F., S.J.B., D.D., C.L.F., D.L., D.T., S.D., J.S.W. Funding acquisition: J.S.W., S.D., D.T., D.L., C.L.F.

## Funding

This work was supported by Simons Foundation, 721231, SFI‐LS‐PROJECT‐00011159, MPS‐SIP‐00930382, 549931, SFI‐LS‐Project‐00011157, SFI‐LS‐Project‐00010539, 721254, SFI‐LS‐Project‐00011154, SFI‐LS‐Project‐01157186, CBIOMES‐00827829; National Science Foundation, 2445508, 2023680; Montgomery County, Maryland; MPower Maryland.

## Conflicts of Interest

The authors declare no conflicts of interest.

## Supporting information


**Table S1:** Parameters associated with the growth of the four phytoplankton types, their respective viruses and zooplankton.
**Table S2:** Default model parameters without environmental effects.
**Table S3:** Factors modulating model parameters to represent different ocean environments for *Prochlorococcus*.
**Table S4:** Ranges and target concentrations for the four phytoplankton types for the two epipelagic ocean types, constraining model testing.
**Figure S1:** Model of burst size as a function of host volume and virion radius for four types of models, optimised using leave one out cross validation.
**Figure S2:** Model versus data of burst size for four types of models, optimised using leave one out cross validation.
**Figure S3:** Model of latent period as a function of host volume and virion radius for four types of models, optimised using leave one out cross validation.
**Figure S4:** Model versus data of latent period for four types of models, optimised using leave one out cross validation.
**Figure S5:** Model output for 5 year time series in nitrogen molar concentrations of the SVZ, SIVZ and the SVRZ models for Prochlorococcus for four different adsorption rates of the virus without quadratic mortality terms.
**Figure S6:** Model output of time series of two example coexistence regime between the virus and the zooplankton in the SIVZ model without quadratic mortality terms of the predators.
**Figure S7:** Growth rate analysis of the free virus in the coexistence regime.
**Figure S8:** Model output of time series of four example coexistence regime between the virus and the zooplankton in the SVRZ model without quadratic mortality terms of the predators.
**Figure S9:** Equilibrium regimes of a virus and a zooplankton modelled as predators of a *Prochlorococcus* for different types of predation models across the adsorption rate and resistant strength parameter space.
**Figure S10:** Simulated and approximate theoretical equilibrium regimes of the SIVZ model for four different phytoplankton with different molar quotas and burst sizes across the spectrum of latent period and adsorption rate of the virus.
**Figure S11:** Equilibrium types for the five different possible equilibria of the SIVZ model in the parameter space for *Prochlorococcus*.
**Figure S12:** Simulated and approximate theoretical equilibrium regimes of the extracellular SIVRZ model for four different phytoplankton with different molar quotas and burst sizes across the spectrum of latent period and adsorption rate of the virus.
**Figure S13:** Equilibrium types for the 11 different possible equilibria of the SIVRZ model in the parameter space for *Prochlorococcus*.
**Figure S14:** Fourier analysis of the virus time series in the last year of simulation for different parameterization of the SIVZ model of Prochlorococcus.
**Figure S15:** Model output of 5 year time series in nitrogen molar concentrations of the SIVZ and the extracellular SIVRZ models for Prochlorococcus for four different adsorption rates of the virus and different resistant type strength.
**Figure S16:** Decomposition of the concentrations of the phytoplankton in the *S*, *I* and *R* cell types in SIV(R)Z models for Prochlorococcus at equilibrium.
**Figure S17:** Distribution of concentration measurements of Prochlorococcus, Synechococcus, their viruses and percentages of infected cells in the North Pacific Subtropical Gyre.
**Figure S18:** Percentage error compared to the target concentration of the different tracers for different models.
**Figure S19:** Decomposition of the conditions imposed on the models' ecology for three different models in the mesotrophic environment.

## Data Availability

All code and data necessary to generate all figures and tables are available at https://github.com/PaulFremont3/SIVZ_coexistence/ and are archived on Zenodo at https://doi.org/10.5281/zenodo.15866512 (Frémont 2025).
